# Identification and distribution of a single nucleotide polymorphism responsible for the catechin content in tea plants

**DOI:** 10.1038/s41438-020-0247-y

**Published:** 2020-03-01

**Authors:** Chen-Kai Jiang, Jian-Qiang Ma, Yu-Fei Liu, Jie-Dan Chen, De-Jiang Ni, Liang Chen

**Affiliations:** 1 Key Laboratory of Tea Biology and Resources Utilization, Ministry of Agriculture and Rural Affairs, Tea Research Institute of the Chinese Academy of Agricultural Sciences, 9 South Meiling Road, Hangzhou, Zhejiang 310008 China; 2 College of Horticulture and Forestry Science, Huazhong Agricultural University, 1 Shizishan Street, Hongshan District, Wuhan, Hubei 430070 China; 3 Tea Research Institute, Yunnan Academy of Agricultural Sciences, Menghai, Yunnan 666201 China

**Keywords:** Secondary metabolism, Transcriptomics

## Abstract

Catechins are the predominant products in tea plants and have essential functions for both plants and humans. Several genes encoding the enzymes regulating catechin biosynthesis have been identified, and the identification of single nucleotide polymorphisms (SNPs) resulting in nonsynonymous mutations within these genes can be used to establish a functional link to catechin content. Therefore, the transcriptomes of two parents and four filial offspring were sequenced using next-generation sequencing technology and aligned to the reference genome to enable SNP mining. Subsequently, 176 tea plant accessions were genotyped based on candidate SNPs using kompetitive allele-specific polymerase chain reaction (KASP). The catechin contents of these samples were characterized by high-performance liquid chromatography (HPLC), and analysis of variance (ANOVA) was subsequently performed to determine the relationship between genotypes and catechin content. As a result of these efforts, a SNP within the *chalcone synthase* (*CHS*) gene was shown to be functionally associated with catechin content. Furthermore, the geographical and interspecific distribution of this SNP was investigated. Collectively, these results will contribute to the early evaluation of tea plants and serve as a rapid tool for accelerating targeted efforts in tea breeding.

## Introduction

Tea, an important healthy beverage that is popular worldwide, is made from fresh leaves harvested from tea plants belonging to the family Theaceae, genus *Camellia* L., section *Thea* (L.) Dyer, including *Camellia sinensis* (L.) O. Kuntze, with the varieties var. *sinensis*, var. *assamica* (Masters) Kitamura, and var. *pubilimba* Chang and the related wild species *C. tachangensis* F. C. Zhang, *C. taliensis* (W. W. Smith) Melchior, *C. crassicolumna* Chang, and *C. gymnogyna* Chang^1^. Among them, *C. sinensis*, the main source of commercially grown tea, is widely cultivated in more than 50 countries^2^. The most extensive genetic variation of tea germplasm worldwide is found in China due to its long history of tea breeding and substantial cultivated genetic resources^3^. These vast genetic resources can serve as an invaluable foundation for modern tea breeding endeavors, and efforts should be taken to ensure that the germplasm is conserved in the future. Currently, the primary objectives for tea breeding are aimed toward the management of early flushing of growth in the spring, special characteristics, high yield, and increased resistance to biotic and abiotic stresses^4^.

Conventional tea breeding is a slow and expensive process owing to the characteristically long juvenile period of tea plants. Typically, at least a 20-year time period is required to complete a cycle of tea breeding, starting from mass selection to local adaptability testing and final release to the public^5^. To date, individual selection and controlled hybridization represent traditional breeding approaches for tea plants. Therefore, novel methods to effectively evaluate and select desirable alleles from existing tea resources are urgently needed to improve the efficiency of tea breeding. Over the last decade, mutation breeding and molecular assisted selection have been further developed with the application of new technologies, especially those related to molecular markers, including restriction fragment length polymorphism (RFLP), amplified fragment length polymorphism (AFLP), simple sequence repeat (SSR), and single nucleotide polymorphism (SNP) analyses. Collectively, these techniques help to efficiently identify and isolate functional sequences with the goal of improving the precision of breeding^6–9^. As described by Lander in 1996^10^, SNPs exhibit a typical mutation rate of ~10^−9^ per nucleotide and have received increasing attention from researchers for their critical role in allele mining, genetic mapping and germplasm identification. Nevertheless, most of the SNPs identified in tea plant are putative^11,12^ and have not been functionally assessed. Specifically, only a few SNPs have been validated and developed into markers for screening tea genetic resources to date.

Flavonoids and their derivatives are a major group of compounds in tea^13^ that are known to confer stress tolerance to tea plants during growth and development^14,15^. Polyphenols are the major flavonoids found within fresh and commercial tea leaves^16^. Catechins, which account for more than 70% of polyphenols^17^, can be divided into ester and nonester catechins^18^. These multifunctional polyphenols help to reduce reactive oxygen species and improve the environmental adaptability of plants^19,20^. In addition, they possess beneficial functions related to the improvement of cardiac function and have anti-inflammatory, antiaging and lipid-lowering effects^21–23^. In tea, the main catechins are epigallocatechin gallate (EGCG), epicatechin gallate (ECG), epigallocatechin (EGC), and epicatechin (EC)^18^. Some key genes in the catechin biosynthesis pathway, including phenylalanine ammonia-lyase (*PAL*), cinnamate 4-hydroxylase (*C4H*), 4-coumarate-CoA ligase (*4CL*), chalcone synthase (*CHS*), chalcone isomerase (*CHI*), flavonoid 3′-hydroxylase (*F3*′*H*), flavonoid 3′5′-hydroxylase (*F3*′*5*′*H*), dihydroflavonol 4-reductase (*DFR*), flavonol synthase (*FLS*), anthocyanidin reductase (*ANR*), and leucoanthocyanidin reductase (*LAR*), have been identified and cloned in tea plants^24^. Alterations in the expression level and mutations of these gene sequences, such as base substitutions, insertions, deletions, and dynamic mutations, affect compound metabolism^25,26^. Regarding the effects on catechin regulation at the gene level, very limited information exists pertaining to the effects of SNPs within gene sequences^27,28^. In the current study, two parents were artificially hybridized to obtain the first filial (F1) offspring. The transcriptomes from two parents and four selected F1 offspring with significant differences in catechins were sequenced and aligned to the reference genome. Twenty-eight candidate SNPs within coding sequences of the highly expressed genes regulating flavonoids were selected for kompetitive allele-specific polymerase chain reaction (KASP). This technical approach aims to identify the SNPs responsible for catechin content. Furthermore, KASP provides insights into the development of SNP markers for the evaluation and selection of unique tea resources, which can contribute to the effective management and genetic improvement of tea plants.

## Results

### Sequencing and mapping

The cDNA libraries were constructed and sequenced by using an Illumina HiSeq 2500 platform. Specifically, transcriptome sequencing data were generated from six samples: ‘Yingshuang’ (YS), ‘Beiyue Danzhu’ (BD), F1-1, F1-2, F1-3, and F1-4. Within the generated datasets, the raw reads ranged from 29.19 to 100.81 million reads, with >97% of the reads passing the quality and trimming filters as described in Table 1. Subsequently, the clean reads were aligned with two published genomes of tea plant, namely, *C. sinensis* var. *sinensis* (CSS)^29^ and *C. sinensis* var. *assamica* (CSA)^30^. The ratio of total mapped reads between each of the six samples and CSS or CSA was 90.77 ~ 92.40% and 83.00 ~ 87.40%, respectively. The mapping information demonstrated that the genetic relationship between the six samples and CSS was more similar than that with CSA. Therefore, the following analysis was based on the sequences that were mapped to the CSS genome.

**Table 1:** Description of the RNA sequencing data and mapping information

Sample		YS	BD	F1-1	F1-2	F1-3	F1-4
Raw reads (×10^6^)		29.19	105.29	90.04	85.57	80.70	100.81
Clean reads percentage (%)		98.39	98.65	97.58	97.52	97.36	97.73
Q20^a^ (%)		95.67	95.51	94.52	94.56	94.36	94.61
Q30^b^ (%)		92.00	91.73	89.52	89.63	89.27	89.69
GC content (%)		46.29	46.33	45.02	44.95	45.65	45.23
Total mapped (%)	CSS	90.77	91.12	92.30	92.13	92.40	91.78
	CSA	83.00	86.52	86.45	87.32	87.40	86.86
Multiple mapped (%)	CSS	7.33	5.73	5.69	5.38	5.44	5.47
	CSA	3.39	3.28	3.35	3.36	3.40	3.39
Uniquely mapped (%)	CSS	83.43	85.39	86.61	86.75	86.95	86.31
	CSA	79.62	83.24	83.11	83.96	84.00	83.47
Nonsplice reads (%)	CSS	56.67	55.86	57.02	56.58	55.99	55.57
	CSA	54.60	55.25	55.41	55.47	54.79	54.49
Splice reads (%)	CSS	26.76	29.52	29.59	30.18	30.97	30.74
	CSA	25.02	27.99	27.70	28.48	29.21	28.97

^a^ and ^b^ represent the percentages of correct base identification that were >99% and 99.9%, respectively

### Analysis of the gene expression levels

**Fig. 1:**
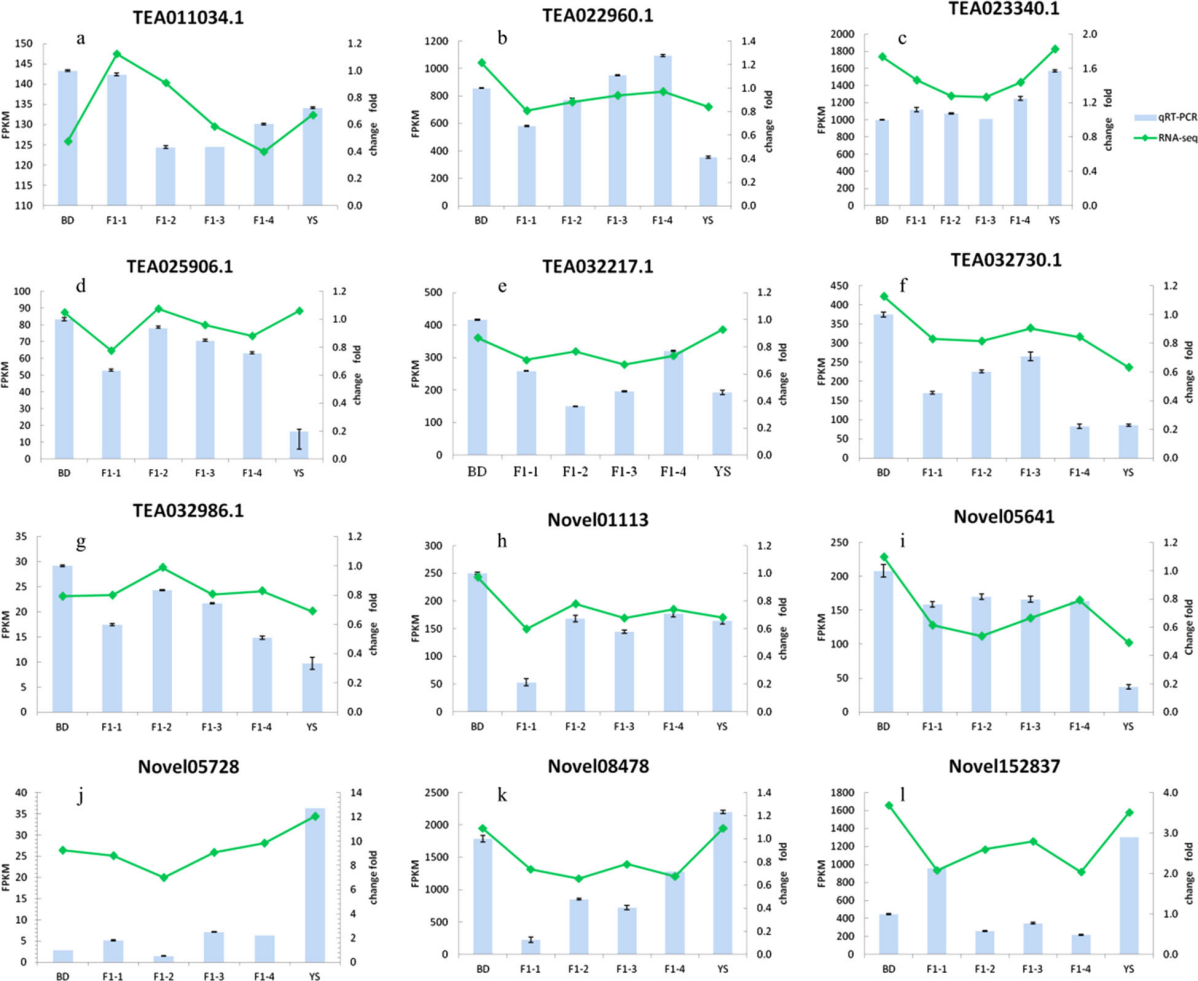
Verification of the relative gene expression levels by qRT-PCR analysis. The expression patterns of 12 genes by qRT-PCR and RNA-seq analyses: **a** TEA011034.1. **b** TEA022960.1. **c** TEA023340.1. **d** TEA025906.1. **e** TEA032217.1. **f** TEA032730.1. **g** TEA032986.1. **h** Novel01113. **i** Novel05641. **j** Novel05728. **k** Novel08478. **l** Novel152837

Twelve genes that were functionally related to metabolism were randomly chosen for quantitative real-time polymerase chain reaction (qRT-PCR) analysis, and their primers are listed in Table S1. The FPKM values were calculated to determine the expression levels based on the number of mapped genes. Collectively, these data revealed that the RNA sequencing results were generally consistent with the qRT-PCR data presented in Fig. 1.

### KEGG enrichment analysis of the DEGs

**Fig. 2:**
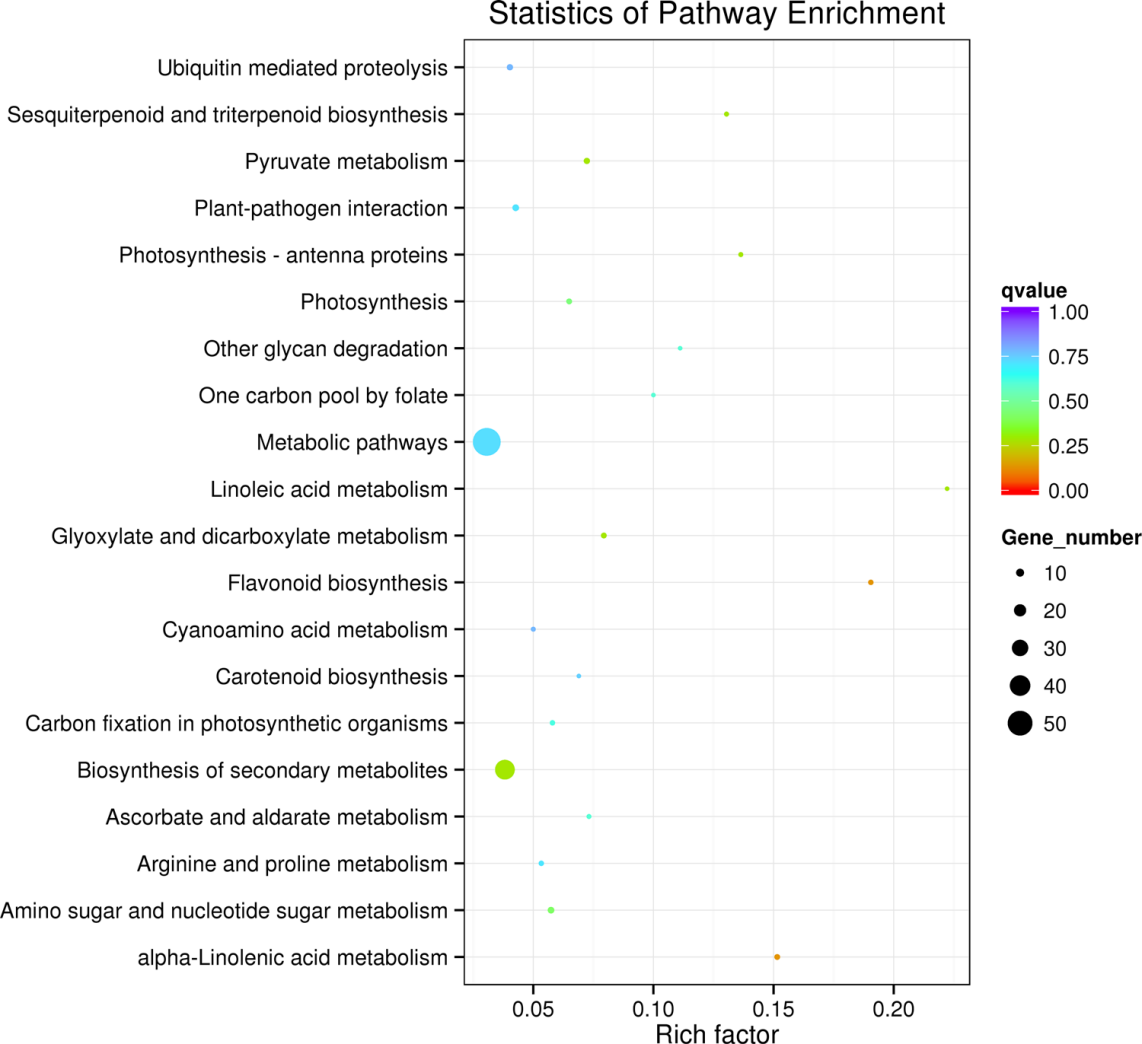
Top 20 enriched KEGG pathways between BD and YS

Differentially expressed genes (DEGs) were identified by comparative analyses between every two samples. Then, a KEGG enrichment analysis was conducted to investigate the functional roles of the identified DEGs. Figure 2 illustrates the top 20 pathway enrichment items of the parents BD and YS, where a decrease in the Q value is directly related to an increased significance of the enrichment. Consequently, “flavonoid biosynthesis” and “alpha-linolenic acid metabolism” were the most notable enriched pathways, with the top two groups of genes being involved with metabolic pathways and the biosynthesis of secondary metabolites. Among these pathways, the flavonoid and phenylpropanoid biosynthetic pathways were of interest for further analysis since catechins are products of phenylpropanoid and flavonoid metabolism. Among the pathways for the upregulated DEGs from the F1-2 vs. BD comparison, “flavonoid biosynthesis” was the most enriched pathway (Fig. S1), whereas “phenylpropanoid biosynthesis” was the most enriched pathway among the pathways for the upregulated DEGs from the BD vs. YS comparison (Fig. S2).

### Detection and functional validation of the SNPs

**Fig. 3:**
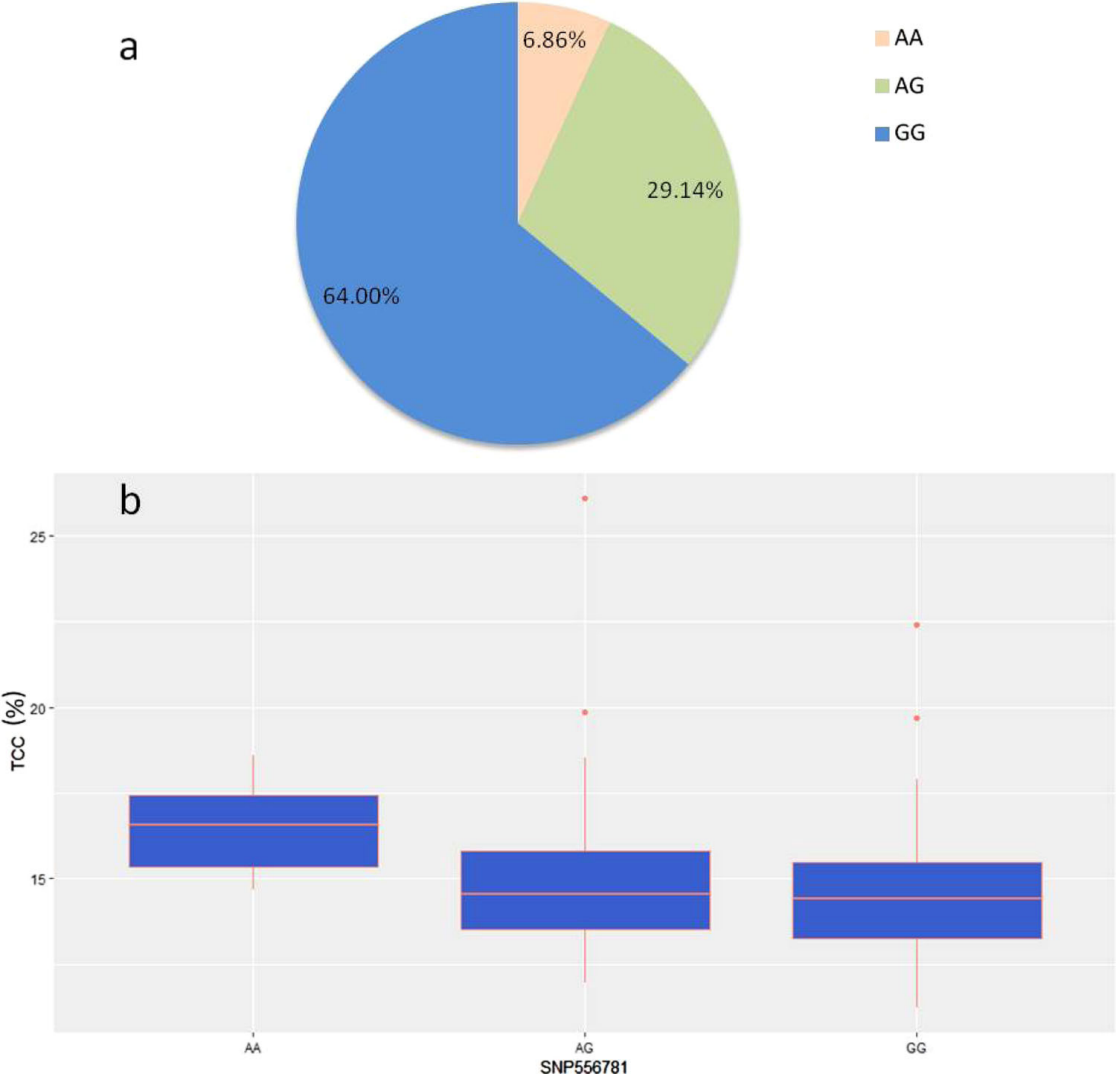
Genotypes of SNP556781 in 176 accessions of tea plants. **a** The percentages of AA, AG, and GG. **b** Total catechin content in tea plants with three different SNP556781 genotypes

The discovery and identification of the SNPs was performed according to the CSS genome. By using a combined analysis of six transcriptomes and a reference genome, we identified a total of 1,376,014 high-quality putative SNPs. In addition to the aforementioned DEGs from the flavonoid and phenylpropanoid biosynthetic pathways, eleven major genes (*PAL*, *C4H*, *4CL*, *CHS*, *CHI*, *F3*′*H*, *F3*′5′*H*, *DFR*, *FLS*, *ANR*, *LAR*) that are associated with flavonoid and phenylpropanoid metabolism on NCBI (https://www.ncbi.nlm.nih.gov/) were identified and aligned to the gene sequences obtained from RNA sequencing data. Then, the SNPs identified within the coding sequences of these genes were further studied by KASP analysis. Twenty accessions were tested two times to examine the reliability of the SNP identification (Table S2). Among the accessions, only one was inconsistent, demonstrating that 95% of the genotypes were reliable. Eventually, SNP556781 was validated and found to regulate the total catechin content (TCC) in tea plants, with three specific genotypes existing (AA, AG, and GG) (Fig. 3a, Tables S2 and S5).

Specifically, a nonsynonymous SNP is located in the coding sequence of TEA023340.1 where the 43rd amino acid is isoleucine (Ile) or threonine (Thr) when A or G is located at the SNP556781 site (Fig. S3a), respectively. The genotype AA only accounted for 6.86%, whereas the GG genotype accounted for up to 64.00%. Thus, we concluded that GG was the dominant genotype at the SNP556781 site.

As shown in Fig. 3b, SNP556781 makes a major contribution to TCC (*p* < 0.01) based on ANOVA (Table S3). The median TCC values of the tea accessions with the genotypes AA, AG, and GG were 16.59%, 14.55%, and 14.41%, respectively. Collectively, these data suggest that the tea plants with the homozygous genotype AA could accumulate substantially more catechin than those with the homozygous genotype GG and heterozygous genotype AG. However, no significant difference in TCC was observed between genotypes AG and GG.

### Genotype distribution of SNP556781

**Fig. 4:**
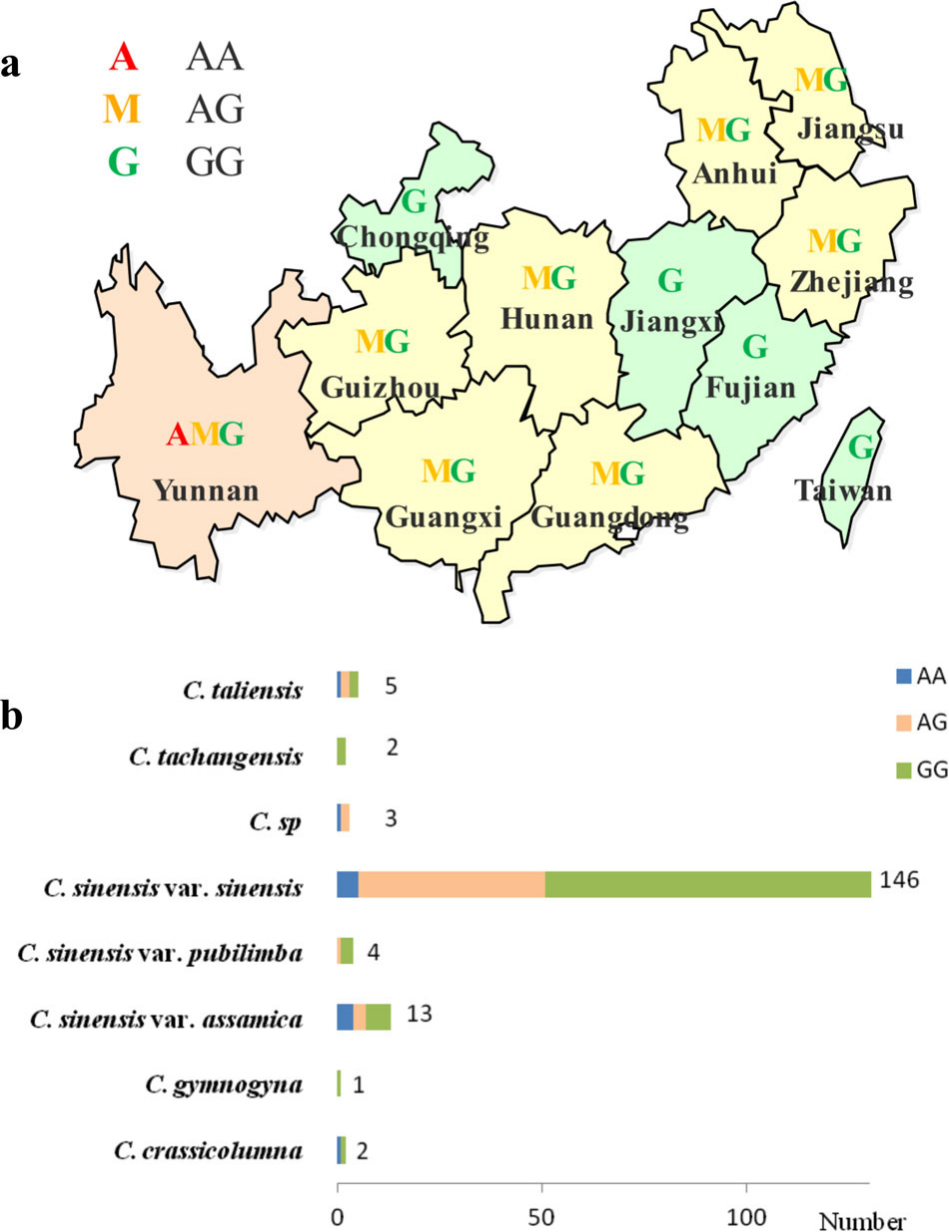
Genotype distribution of SNP556781 in the core tea germplasms. **a** Geographical distribution of the genotypes. **b** Distribution of the genotypes in different species and varieties (the number represents the sum accession of each species and variety)

As shown in Fig. 4a, genotype AA only appears in Yunnan, which was the origin center of the tea plant^31^, implying that AA was the primitive genotype. Tea plants from Yunnan, Guizhou, Guangxi, Hunan, Guangdong, Anhui, Zhejiang, and Jiangsu possessed the genotype AG. A distinctive genotype constitution was observed in a different group of *Thea* sections (Fig. 4b). Specifically, *C. taliensis*, *C. sinensis* var. *sinensis*, and *C. sinensis* var. *assamica* had three genotypes. Nevertheless, it was difficult to determine whether the remaining groups lacked one or two genotypes, since insufficient samples were available for the analysis, with the exception of *C. sinensis* var. *sinensis*. In addition, all *C. sinensis* var. *sinensis* cultivars with the AA genotype are F1 individuals that are the offspring of YS and BD.

## Discussion

SNPs are becoming an increasingly popular and powerful tool to enable a genetic analysis of the functional mechanisms in tea plants, and research on this topic has resulted in significant achievements in the last 5 years. For example, large-scale SNPs combined with SSRs in an F1 population (YS × BD) were used for the construction of the first high-density genetic map of tea plant in 2015^32^. Then, a high-density SNP linkage map was constructed, and QTLs related to flavonoids that were dependent on the F1 population (LJ43 × BHZ) were identified in 2018^12^. Candidate QTLs and proteins were shown to be involved in flavonoid biosynthesis using DArTseq markers via two F1 populations (TRFK 303/577 × GW Ejulu and GW Ejulu × TRFK 303/577) in 2018 and 2019^33,34^. SNPs and InDels were identified based on whole genome sequences of ‘Shuchazao’ and ‘Yunkang 10’ in 2019. Many of these SNPs/InDels were in catechin/caffeine biosynthesis-related genes, although they have not been validated^35^.

Although some QTLs related to flavonoids were identified in these previous studies, they have not been further validated. As a result, it is still difficult to take full advantage of the putative QTLs in molecular marker-assisted breeding efforts. In the present study, we aimed to identify novel SNPs by adopting a new strategy. First, precise information to enable SNP discovery was obtained by using the reference genomes of tea plant. Second, candidate SNPs selected from RNA sequencing data obtained from test groups were further explored by KASP. Finally, the verification group was more representative and comprehensive than the previous group since it contained natural populations in addition to an F1 population. Collectively, the results showed that this strategy is an economic and effective way to mine SNPs that are functionally related to catechin content.

SNPs affect the functions of genes via nonsynonymous mutations or by regulating expression levels. The SNP position in the gene sequence controls its effect on TCC. For example, TEA023340.1 is located within scaffold2074: 55434-55909, which contains the sequence encoding CHS (EC 2.3.1.74). CHS is the rate-limiting enzyme of flavonoid biosynthesis in the general phenylpropanoid pathway^36^. Specifically, CHS is the first committed enzyme of flavonoid biosynthesis and catalyzes the stepwise condensation of three acetate residues from malonyl-CoA with the phenylpropanoid biosynthetic intermediate p-coumaryl CoA to form naringenin chalcone, which leads to the synthesis of various flavonoid derivatives^37^. By using a BLAST analysis in the Protein Data Bank, *CHS* in *C. sinensis* was shown to be most similar to chain A of *CHS* (PDB ID: 5CU5) in apple, with a similarity of up to 99% with *p* = 0.00. The threonine, serine and tyrosine residues are likely sites for modification by phosphorylation^38^. According to predictive analysis (http://www.dabi.temple.edu/disphos/), the 43rd Thr residue is not a phosphorylation modification site with a G allele at SNP556781 (Fig. S3b). The replacement of a solvent-exposed hydrophobic residue (Ile) with a hydrophilic residue (Thr) might impart thermostability to the protein structure^39^. Therefore, this finding implies that the genotypes GG and AG at SNP556781 could enhance the stability of CHS relative to the genotype AA. However, tea plants with genotype AA have higher TCC values than those with genotypes GG and AG. In addition to amino residues, spatial conformation plays a critical role in the stability and activity of proteins. Further evaluations are warranted to explore the contributions of particular SNP mutations to the characteristics of proteins. Moreover, additional investigations of the metabolic mechanisms, especially upstream and downstream metabolites of catechins, are warranted and necessary to find genes that are closely related to *CHS*.

The regulation of traits can occur by a series of very complicated steps, and some are controlled by minor genes, with single genes that may possess multiple effects. As an illustration, *qγ27* in maize resulted from a 15.26 kb duplication at the 27-kDa γ-zein locus, which contains four genes. Genotypic results showed that this duplication significantly influenced the Lys/total levels and the expression levels of the four genes^40^. In accordance with these observations, the function of SNP556781 should be further investigated by an analysis that integrates other phenotypes. Furthermore, the construction of a coexpression network is a powerful strategy that can be employed for detecting the relationships among various genes and to gain a better understanding of the regulatory principles.

In the current study, the tea plants and the wild relatives used were native to the main tea production area in China, and only tea plants from Yunnan possessed the genotype AA. In other words, they included all three genotypes, indicating that there is rich genetic diversity in Yunnan. Consequently, it can be implied that the tea plant and its relatives in Yunnan preserve the most alleles as the most important reservoirs of genetic variation, which is in accordance with the results from Yao et al.^3^, Fang et al.^41^, and Huang et al.^42^. The domestication effects may partially explain the higher frequency of the AA genotype, since TCC was negatively selected during domestication, in exchange for improved taste. TCC was affected by both genetic background and environment. Generally, TCC increases with higher temperature and stronger sunlight^42^. The TCC of tea plants decreased northward and eastward from the original location. Therefore, a decrease in the TCC is the result of both domestication and environmental effects. To effectively study the genetic mechanism and protection of tea plants, researchers should take full advantage of the existing natural resources for tea plants. Furthermore, additional resources are required to explore the exhaustive genotypic distribution in diverse species, since the majority of plants were *C. sinensis* var. *sinensis* in the current study.

No significant difference was found among the TCCs of the samples with the genotypes AG and GG. The TCC in tea plants with AA at SNP556781 was obviously higher than that observed in the plants with the genotypes AG and GG. It is likely that AA promotes the biosynthesis or inhibits the degradation of catechin and that its genotype-dependent capacity is genotype dependent. In conventional tea plants, flavonoid metabolism is enhanced under strong light, but the opposite results are observed in light-sensitive tea plants^19^. Catechins are usually decreased in green-leaf tea plants with shading treatment due to an alternation in the transcription level of the genes responsible for catechin biosynthesis^43^. In the present study, tea plants were grown in the same surroundings. Consequently, we concluded that the observed variation of DNA sequences, rather than the cultivation environment, is the contributing factor that influences catechin metabolism for these experimental plants. Moreover, not all the cultivars with the highest TCC values had the AA genotype, which suggests that other genes may also regulate TCC. A multi-environmental trial would further improve the validity of our findings, which deserves further analysis.

## Materials and methods

### Plant materials

A controlled hybridization population with approximately 300 individuals was generated for this study. The male parent was ‘Beiyue Danzhu’ (BD, *C. sinensis* var. *pubilimba*), the landrace in Longzhou of Guangxi. The female parent selected for this study was ‘Yingshuang’ (YS, *C. sinensis* var. *sinensis*), which represents the national approved cultivar selected from natural hybrids between ‘Fuding Dabaicha’ (*C. sinensis* var. *sinensis*) and ‘Yunnan Dayezhong’ (*C. sinensis* var. *assamica*). Four offspring of the F1 population with relatively high or low TCC values named F1-1, F1-2, F1-3, and F1-4 were selected from the ‘BD’ × ‘YS’ population. The abovementioned tea plants and 170 other accessions that are representative of the core collection of tea germplasm^27^ (Tables S4 and S5) were planted and maintained through similar horticultural practices in the China National Germplasm Hangzhou Tea Repository located at the Tea Research Institute, Chinese Academy of Agricultural Sciences. Three biological replicates were harvested at the “two leaves and a bud” stage in April 2018, and the samples were immediately flash frozen with liquid nitrogen and stored at −80 °C until further use.

### Catechin detection

The samples were pretreated and examined as described in a previous study^27^. Specifically, the chromatographic peaks were identified by UV spectroscopy using a diode array detector, and the retention times were compared with those of the authentic standards.

### RNA and DNA extraction

Total RNA was isolated using the RNAprep Pure Plant kit (Tiangen Biotech Co., Ltd., China) according to the manufacturer’s instructions. The purity and degree of degradation of the RNA samples were examined by using a Nanodrop (Thermo Fisher Scientific, USA) and agarose gel electrophoresis, respectively. Then, the RNA samples were quantified using a Qubit RNAssay Kit (Qubit 2.0, USA), and the RNA integrity was assessed by using an Agilent RNA 6000 Pico Assay Kit (Agilent 2100, USA). Samples with RNA integrity values >8.0 along with a ratio of 1.9 ~ 2.1 (A260/A280) and a ratio of 2.0 ~ 2.5 (A260/A230) were selected for sequence analysis. Total DNA was isolated using a DNAsecure Plant Kit (Tiangen Biotech Co., Ltd., China) according to the manufacturer’s instructions.

### Construction of a cDNA library and sequencing

High-quality RNA samples from tea plants were prepared using an Illumina TruSeq RNA Sample Prep Kit (USA), and the cDNA libraries were constructed with an Ultra RNA Library Prep Kit for Illumina (USA). The cDNAs were purified using Beckman AMPure XP beads (USA) and subsequently assessed with an Agilent High Sensitivity DNA Kit (Agilent 2100, USA) for the detection of inserted cDNA fragments. Then, the cDNA libraries were quantified with a Bio-Rad KIT iQ SYBR Green kit (Bio-Rad CFX 96, USA) and subsequently sequenced using a TruSeq SBS Kit v3 (Illumina HiSeq2500, USA).

### Data filtering and mapping to the reference genome

Clean reads were obtained after trimming and eliminating the adapter, ambiguous reads (‘N’) and low-quality reads (bases with Qphred ≤ 20 more than 50% of total bases) with the FastX-toolkit based on Q30. The clean reads were then subsequently aligned to two reference genomes (http://pcsb.ahau.edu.cn:8080/CSS/ and http://www.plantkingdomgdb.com/tea_tree/)^29,30^. An index of the reference genome was built using Bowtie v2.2.3, and paired-end clean reads were aligned to the reference genome using TopHat v2.0.12. A database of splice junctions was generated using TopHat based on the gene model annotation for an optimized mapping result.

### Gene expression analyses

The reads were mapped to each gene using HTSeq, and fragments per kilobase of exon model per million mapped reads (FPKM) were calculated on the basis of the length of the gene and read counts mapped to the reference genes. Differential expression analyses between two samples were performed based on negative binomial distribution through the DESeq R package (1.20.0). In addition, the *p* values were adjusted by using Benjamini and Hochberg’s approach. The differentially expressed genes (DEGs) were identified by DESeq, and the thresholds of the adjusted *p* value and log2 (fold change) were 0.005 and 1, respectively.

### Quantitative real-time PCR (qRT-PCR) analyses

A total of 12 transcripts were randomly selected for validation of gene expression by using quantitative RT-PCR (qRT-PCR). The cDNAs were reverse transcribed using the PrimeScript^TM^ RT reagent qPCR kit (Tiangen Biotech Co., Ltd., China), and the qRT-PCR reactions were conducted using the following parameters: 95 °C for 30 s and 40 cycles at 94 °C for 15 s and 60 °C for 5 s. Three independent biological replicates and three technical replicates of each reaction were performed using GAPDH as a reference gene. Fluorescence intensity was measured through the Applied Biosystems 7500 Sequence Detection System (Carlsbad, CA, USA), and the relative expression values of the genes were subsequently calculated based on the 2^−ΔΔCt^ method.

### KEGG enrichment analysis of the DEGs

The Kyoto Encyclopedia of Genes and Genomes (KEGG) enrichment analysis of the DEGs was implemented using KOBAS 2.0. The significance of the differential expression was assessed by relying on hypergeometry according to the following formula: $$p = 1 - \mathop {\sum }\nolimits_{i = 0}^{m - 1} \frac{{\left( {\begin{array}{*{20}{c}} M \\ i \end{array}} \right)\left( {\begin{array}{*{20}{c}} {N - M} \\ {n - i} \end{array}} \right)}}{{\left( {\begin{array}{*{20}{c}} N \\ n \end{array}} \right)}}$$. In this formula, *n*, *M*, and *m* represent the number of DEGs, genes in one pathway, and DEGs in one pathway, respectively. The DEGs were substantially enriched in the KEGG pathway with a false discovery rate of <0.05.

### SNP validation

The DEGs were aligned to the genes that were responsible for flavonoid metabolism. The matched DEGs that had an FPKM >1000 were used for SNP screening, and 28 SNPs in the coding regions of the genes were selected for functional investigation. These SNPs were tested in 176 tea accessions by using KASP, and 20 accessions were randomly selected for two replicate analyses. The SNPs and primers that were used for KASP are displayed in Table S1. The tea accessions were grouped based on SNP types, and their catechin contents were compared using analysis of variance (ANOVA) at a threshold of *p* < 0.05. The results were subsequently analyzed and visualized by using R 3.5.1.

## Supplementary information

Supplemental materials
