# Supplementary material for: Identification and distribution of a single nucleotide polymorphism responsible for the catechin content in tea plants
Source: Hortic Res. 2020 Mar 1;7:24. doi: 10.1038/s41438-020-0247-y (PMC7049304; doi:10.1038/s41438-020-0247-y)
Supplement: Supplementary file 1 — Supplemental materials [file 41438_2020_247_MOESM1_ESM.docx]

Supplemental materials

Table S1 Information of PCR and KASP

| \| \| PCR primer \| \| \| --- \| --- \| \| 5‘-3’ \| 3‘-5’ \| \| ACCCAACACGATGAGACCG \| GCCAATGACAAAGCGACCA \| \| ATGGAGTTTTGTCACGAAAATGG \| TTAACAGATTGGAAGAGGAGCACC \| \| ATGGTCACCGTCGAGGACATCCG \| TTATGTAGACAAGCTATGGAGCAC \| \| ATGGAGAACCAAAAACAGAAAC \| TTAATTGGCAAAGCCAGCTGCG \| \| TCTTTCTGGACCTGTGAGTG \| GCTTGAGGGTAGATAATGAGT \| \| ATGAAAGACTCTGTTGCTTCTGC \| TTAAACCTTGTTGCCATTGACAG \| \| ATGGCGCCAACAACAACGCTTAC \| TCAAGCAAAAATCTCATCAGTGC \| \| ATGGCTTGCGGTACTCTG \| ATTCGGATGGTGTTGTCG \| \| GTCACAACCACCAAGCCTCTC \| GCCCTTCCATTGCTGCAA \| \| ATGGAGAATGCTAATGAGAGCT \| TTAACAAATTGGAAGAGGTGCACC \| \| GCTGTCGAGATGGGTATCA \| GCCGAGAACAACAAAGGA \| \| TGTGGGTCAAGCCCTATTT \| AACGGTTTCTCAACTTCTGG \| \| \| --- \| --- \| --- \| --- \| --- \| --- \| --- \| --- \| --- \| --- \| --- \| --- \| --- \| --- \| --- \| --- \| --- \| --- \| --- \| --- \| --- \| --- \| --- \| --- \| --- \| --- \| --- \| --- \| --- \| \|  \| \| \| **KASP** \| FAM \| VIC \| COM \| Position \| mutation \| \| --- \| --- \| --- \| --- \| --- \| --- \| \| CsSNP77431 \| GCATAACAGGAAGTTAGCAAGCACG \| CATAACAGGAAGTTAGCAAGCACA \| CTCTCCCTTCTGTTGAGCATCCAAA \| 77431 \| [G/A] \| \| CsSNP77448 \| AAGCACRAAGAGCATGGACAACAA \| GCACRAAGAGCATGGACAACAG \| GTTGTCCTCGTTGATCTCTCCCTTY \| 77448 \| [A/G] \| \| CsSNP77491 \| GATTGATCATATTTTGGATGCTCAACAG \| CGATTGATCATATTTTGGATGCTCAACAA \| CAGCGACGTTAATGTTCTCSACGAT \| 77491 \| [G/A] \| \| CsSNP77530 \| AATACCAGCGACGTTAATGTTCTCG \| AATACCAGCGACGTTAATGTTCTCC \| GAGAGATCAACGAGGACAACGTTCTT \| 77530 \| [C/G] \| \| CsSNP80253 \| CTTGACACCATGCTTGGCCC \| AACTTGACACCATGCTTGGCCT \| TTGTAGGTGTCTGGCTCGGTGATTT \| 80253 \| [C/T] \| \| CsSNP80722 \| CAGAACGATCTTGGTTTCAGAACG \| AAATCAGAACGATCTTGGTTTCAGAACA \| GGGGGACAGTTCAGCTTGCATATTT \| 80722 \| [C/T] \| \| CsSNP80848 \| TCTGAATTGTCCTTGCACATCAAC \| AACTTCTGAATTGTCCTTGCACATCAAT \| GGTTGAGAAGGAATTGGGGAGATGTT \| 80848 \| [G/A] \| \| CsSNP80894 \| AACTGGTAATCACATACAGAGACAT \| ACTGGTAATCACATACAGAGACAG \| GGACAATTCAGAAGTTTGCAAGGAGTATT \| 80894 \| [A/C] \| \| CsSNP104938 \| CTCTCTCTCTCTCAATGGAGA \| TCTCTCTCTCTCTCAATGGAGG \| GGAGTGGCAAGTGATTTGGGATGTA \| 104938 \| [A/G] \| \| CsSNP105129 \| GAGGTTGTTGAGGCCGGCG \| GAGGTTGTTGAGGCCGGCC \| AATTCAGCTCTCGTCCCTGCATCAT \| 105129 \| [C/G] \| \| CsSNP106077 \| CCATCGTGCTGGCGATCGCA \| CATCGTGCTGGCGATCGCG \| CCGTCCGAATCGACGACAAATCATA \| 106077 \| [A/G] \| \| CsSNP108375 \| GTGGGACTGTTGTCAGAAATGCC \| ATGTGGGACTGTTGTCAGAAATGCT \| CAGTGTCAGGGTCTACGATCTTCAT \| 108375 \| [C/T] \| \| CsSNP108450 \| GGAGAAATTTGCATCAGGGGT \| CTGGAGAAATTTGCATCAGGGGC \| CGGGTATGGGCATACACTAGTGTAT \| 108450 \| [T/C] \| \| CsSNP487072 \| ATGTAGCTAGATTATGAAGCAGAGGA \| GTAGCTAGATTATGAAGCAGAGGC \| GGTTTATGTGAGTGCTTAAGTTGCTGAAA \| 487072 \| [A/C] \| \| CsSNP487090 \| AAGCAGAGGMAGAACATAAATATAGCAT \| AAGCAGAGGMAGAACATAAATATAGCAA \| GCTGCTGCTGCTGCTGAATAAGTAT \| 487090 \| [T/A] \| \| CsSNP487142 \| TTTTCAGCAACTTAAGCACTCACA \| TTTTTCAGCAACTTAAGCACTCACG \| GCTGCTGCTGCTGCTGAATAAGTAT \| 487142 \| [A/G] \| \| CsSNP488478 \| GGAAAGTTTCATCCTTGAAGACCTCA \| GAAAGTTTCATCCTTGAAGACCTCG \| GATGCAGAGGCCAAAGCCATTGAAA \| 488478 \| [A/G] \| \| CsSNP490956 \| CAAACAGTTGTTACAAGAACAAGCG \| CCAAACAGTTGTTACAAGAACAAGCA \| CTATGTGCCCAGTCTAAATGGCCAT \| 490956 \| [G/A] \| \| CsSNP491027 \| GTCTTGAGATAAGAAGGAATGAACAGA \| CTTGAGATAAGAAGGAATGAACAGG \| GCACCTCTGATATTTTCACCGGCAA \| 491027 \| [A/G] \| \| CsSNP491084 \| AGAGGTGCGGTAGTTGGAGC \| CAGAGGTGCGGTAGTTGGAGT \| CAAACTCACCAGCCCACAAACCTAA \| 491084 \| [C/T] \| \| CsSNP491185 \| ACTTCCCAACTCCTGGATGACTTA \| CTTCCCAACTCCTGGATGACTTG \| GAATTAGGTTTGTGGGCTGGTGAGTT \| 491185 \| [T/C] \| \| CsSNP491247 \| GGGTCAGCTGGCAAGAGATG \| TTGGGTCAGCTGGCAAGAGATA \| CAGTATCAACTGCATATGCSGCCAT \| 491247 \| [G/A] \| \| CsSNP491296 \| CAGCAGTATCAACTGCATATGCC \| CAGCAGTATCAACTGCATATGCG \| GTCAGCTGGCAAGAGATRTGGACTA \| 491296 \| [G/C] \| \| CsSNP555338 \| TGTTTGGTTCTACTTTATTTCCTGCA \| TTTGTTTGGTTCTACTTTATTTCCTGCT \| CCCAAGATTAATTGTCTCATTTTTTACCAT \| 555338 \| [T/A] \| \| CsSNP555403 \| ACACAATCACAAATTGAAGAAAACCCC \| ATAACACAATCACAAATTGAAGAAAACCCA \| GTGGTGCTCCATAGCTTGTCWACAT \| 555403 \| [C/A] \| \| CsSNP555440 \| CAATAGGCTAGCAGCCATTTTATGTA \| CAATAGGCTAGCAGCCATTTTATGTT \| TTGAGACTGTGGTGCTCCATAGCTT \| 555440 \| [A/T] \| \| CsSNP556781 \| GGCCTTATGCTCGCTGTTTGTG \| CGGCCTTATGCTCGCTGTTTGTA \| GATCAGAGCACGTACCCGGATTATT \| 556781 \| [G/A] \| \| CsSNP556898 \| CCGGCCAAAATGGTCACCGTC \| CCGGCCAAAATGGTCACCGTG \| GTGGCAGGTCCTTCAGCTCGTT \| 556898 \| [G/C] \|   Table S2 Genotype of 176 tea accessions   \| Number \| Species \| Origin \| Genotype \| \| --- \| --- \| --- \| --- \| \| SPM001 \| *C. sinensis* (L.) O. Kuntze var*. sinensis* \| Fujian \| GG \| \| SPM002 \| *C. sinensis* (L.) O. Kuntze var*. sinensis* \| Hunan \| GG \| \| SPM003 \| *C. sinensis* (L.) O. Kuntze var*. sinensis* \| Fujian \| GG \| \| SPM004 \| *C. sinensis* (L.) O. Kuntze var*. sinensis* \| Fujian \| GG \| \| SPM005 \| *C. sinensis* (L.) O. Kuntze var*. sinensis* \| Zhejiang \| GG \| \| SPM006 \| *C. sinensis* (L.) O. Kuntze var*. sinensis* \| Fujian \| GG \| \| SPM007 \| *C. sinensis* (L.) O. Kuntze var*. sinensis* \| Fujian \| GG \| \| SPM008 \| *C. sinensis* (L.) O. Kuntze var*. sinensis* \| Fujian \| GG \| \| SPM009 \| *C. sinensis* (L.) O. Kuntze var*. sinensis* \| Fujian \| GG \| \| SPM010 \| *C. sinensis* (L.) O. Kuntze var*. sinensis* \| Zhejiang \| GG \| \| SPM011 \| *C. sinensis* (L.) O. Kuntze var*. sinensis* \| Fujian \| GG \| \| SPM012 \| *C. sinensis* (L.) O. Kuntze var*. sinensis* \| Hunan \| GG \| \| SPM013 \| *C. sinensis* (L.) O. Kuntze var*. sinensis* \| Fujian \| GG \| \| SPM014 \| *C. sinensis* (L.) O. Kuntze var*. sinensis* \| Fujian \| GG \| \| SPM015 \| *C. sinensis* (L.) O. Kuntze var*. sinensis* \| Zhejiang \| GG \| \| SPM016 \| *C. sinensis* (L.) O. Kuntze var*. sinensis* \| Fujian \| GG \| \| SPM017 \| *C. sinensis* (L.) O. Kuntze var*. sinensis* \| Zhejiang \| GG \| \| SPM018 \| *C. sinensis* (L.) O. Kuntze var*. sinensis* \| Zhejiang \| GG \| \| SPM019 \| *C. sinensis* (L.) O. Kuntze var*. sinensis* \| Fujian \| GG \| \| SPM020 \| *C. sinensis* (L.) O. Kuntze var*. sinensis* \| Fujian \| GG \| \| SPM021 \| *C. sinensis* (L.) O. Kuntze var*. sinensis* \| Fujian \| GG \| \| SPM022 \| *C. sinensis* (L.) O. Kuntze var*. sinensis* \| Zhejiang \| AG \| \| SPM023 \| *C. sinensis* (L.) O. Kuntze var*. sinensis* \| Fujian \| GG \| \| SPM024 \| *C. sinensis* (L.) O. Kuntze var*. sinensis* \| Zhejiang \| GG \| \| SPM025 \| *C. sinensis* (L.) O. Kuntze var*. sinensis* \| Fujian \| GG \| \| SPM026 \| *C. sinensis* (L.) O. Kuntze var*. sinensis* \| Zhejiang \| GG \| \| SPM027 \| *C. sinensis* (L.) O. Kuntze var*. sinensis* \| Fujian \| GG \| \| SPM028 \| *C. sinensis* (L.) O. Kuntze var*. sinensis* \| Zhejiang \| GG \| \| SPM029 \| *C. sinensis* (L.) O. Kuntze var*. sinensis* \| Zhejiang \| GG \| \| SPM030 \| *C. sinensis* (L.) O. Kuntze var*. sinensis* \| Zhejiang \| GG \| \| SPM031 \| *C. sinensis* (L.) O. Kuntze var*. sinensis* \| Zhejiang \| GG \| \| SPM032 \| *C. sinensis* (L.) O. Kuntze var*. sinensis* \| Zhejiang \| GG \| \| SPM033 \| *C. sinensis* (L.) O. Kuntze var*. sinensis* \| Jiangxi \| GG \| \| SPM034 \| *C. sinensis* (L.) O. Kuntze var*. sinensis* \| Zhejiang \| GG \| \| SPM035 \| *C. sinensis* (L.) O. Kuntze var*. sinensis* \| Jiangsu \| AG \| \| SPM036 \| *C. sinensis* (L.) O. Kuntze var*. sinensis* \| Jiangsu \| GG \| \| SPM037 \| *C. sinensis* (L.) O. Kuntze var*. sinensis* \| Jiangxi \| GG \| \| SPM038 \| *C. sinensis* (L.) O. Kuntze var*. sinensis* \| Zhejiang \| GG \| \| SPM039 \| *C. sinensis* (L.) O. Kuntze var*. sinensis* \| Zhejiang \| GG \| \| SPM040 \| *C. sinensis* (L.) O. Kuntze var*. sinensis* \| Zhejiang \| GG \| \| SPM041 \| *C. sinensis* (L.) O. Kuntze var*. sinensis* \| Zhejiang \| GG \| \| SPM042 \| *C. sinensis* (L.) O. Kuntze var*. sinensis* \| Zhejiang \| GG \| \| SPM043 \| *C. sinensis* (L.) O. Kuntze var*. sinensis* \| Zhejiang \| AG \| \| SPM044 \| *C. sinensis* (L.) O. Kuntze var*. sinensis* \| Zhejiang \| AG \| \| SPM045 \| *C. sinensis* (L.) O. Kuntze var*. sinensis* \| Zhejiang \| GG \| \| SPM046 \| *C. sinensis* (L.) O. Kuntze var*. sinensis* \| Zhejiang \| AG \| \| SPM047 \| *C. sinensis* (L.) O. Kuntze var*. sinensis* \| Zhejiang \| AG \| \| SPM048 \| *C. sinensis* (L.) O. Kuntze var*. sinensis* \| Zhejiang \| GG \| \| SPM049 \| *C. sinensis* (L.) O. Kuntze var*. sinensis* \| Zhejiang \| GG \| \| SPM050 \| *C. sinensis* (L.) O. Kuntze var*. sinensis* \| Zhejiang \| GG \| \| SPM051 \| *C. sinensis* (L.) O. Kuntze var*. sinensis* \| Jiangxi \| GG \| \| SPM052 \| *C. sinensis* (L.) O. Kuntze var*. sinensis* \| Zhejiang \| GG \| \| SPM053 \| *C. sinensis* (L.) O. Kuntze var*. sinensis* \| Jiangxi \| AG \| \| SPM054 \| *C. sinensis* (L.) O. Kuntze var*. sinensis* \| Zhejiang \| AG \| \| SPM055 \| *C. sinensis* (L.) O. Kuntze var*. sinensis* \| Jiangxi \| GG \| \| SPM056 \| *C. sinensis* (L.) O. Kuntze var*. sinensis* \| Zhejiang \| AG \| \| SPM057 \| *C. sinensis* (L.) O. Kuntze var*. sinensis* \| Zhejiang \| AG \| \| SPM058 \| *C. sinensis* (L.) O. Kuntze var*. sinensis* \| Guangxi \| GG \| \| SPM059 \| *C. sinensis* (L.) O. Kuntze var*. sinensis* \| Hunan \| AG \| \| SPM060 \| *C. sinensis* (L.) O. Kuntze var*. sinensis* \| Jiangsu \| AG \| \| SPM061 \| *C. sinensis* (L.) O. Kuntze var*. sinensis* \| Jiangsu \| AG \| \| SPM062 \| *C. sinensis* (L.) O. Kuntze var*. sinensis* \| Jiangsu \| GG \| \| SPM063 \| *C. sinensis* (L.) O. Kuntze var*. sinensis* \| Jiangsu \| GG \| \| SPM064 \| *C. sinensis* (L.) O. Kuntze var*. sinensis* \| Jiangsu \| GG \| \| SPM065 \| *C. sinensis* (L.) O. Kuntze var*. sinensis* \| Jiangsu \| GG \| \| SPM066 \| *C. sinensis* (L.) O. Kuntze var*. sinensis* \| Jiangsu \| AG \| \| SPM067 \| *C. sinensis* (L.) O. Kuntze var*. sinensis* \| Hunan \| GG \| \| SPM068 \| *C.* sp \| Guangdong \| GG \| \| SPM069 \| *C.sinensis* var. *pubilimba* Chang \| Guangxi \| GG \| \| SPM070 \| *C. crassicolumna* Chang \| Yunnan \| AA \| \| SPM071 \| *C. gymnogyna* Chang \| Yunnan \| GG \| \| SPM072 \| *C. sinensis* var. *assamica* (Masters) Kitamura \| Yunnan \| AA \| \| SPM073 \| *C. sp* \| Yunnan \| GG \| \| SPM074 \| *C. sp* \| Yunnan \| AA \| \| SPM075 \| *C. sinensis* var. *assamica* (Masters) Kitamura \| Yunnan \| AG \| \| SPM076 \| *C. taliensis* (W. W. Smith) Melchior \| Yunnan \| AG \| \| SPM077 \| *C. sinensis* (L.) O. Kuntze var*. sinensis* \| Yunnan \| GG \| \| SPM078 \| *C. sinensis* (L.) O. Kuntze var*. sinensis* \| Yunnan \| AG \| \| SPM079 \| *C. sinensis* var. *assamica* (Masters) Kitamura \| Yunnan \| AG \| \| SPM080 \| *C. taliensis* (W. W. Smith) Melchior \| Yunnan \| AA \| \| SPM081 \| *C. taliensis* (W. W. Smith) Melchior \| Yunnan \| AG \| \| SPM082 \| *C. sinensis* var. *assamica* (Masters) Kitamura \| Yunnan \| AA \| \| SPM083 \| *C. sinensis* var. *assamica* (Masters) Kitamura \| Yunnan \| GG \| \| SPM084 \| *C. sinensis* (L.) O. Kuntze var*. sinensis* \| Yunnan \| AA \| \| SPM085 \| *C. sinensis* var. *assamica* (Masters) Kitamura \| Yunnan \| AA \| \| SPM086 \| *C. crassicolumna* Chang \| Yunnan \| GG \| \| SPM087 \| *C. sinensis* var. *assamica* (Masters) Kitamura \| Yunnan \| AA \| \| SPM088 \| *C. sinensis* (L.) O. Kuntze var*. sinensis* \| Yunnan \| AG \| \| SPM089 \| *C. sinensis* (L.) O. Kuntze var*. sinensis* \| Yunnan \| AG \| \| SPM090 \| *C. sinensis* (L.) O. Kuntze var*. sinensis* \| Yunnan \| GG \| \| SPM091 \| *C. tachangensis* F. C. Zhang \| Guizhou \| GG \| \| SPM092 \| *C. tachangensis* F. C. Zhang \| Guizhou \| GG \| \| SPM093 \| *C. taliensis* (W. W. Smith) Melchior \| Yunnan \| GG \| \| SPM094 \| *C. taliensis* (W. W. Smith) Melchior \| Yunnan \| GG \| \| SPM095 \| *C. sinensis* (L.) O. Kuntze var*. sinensis* \| Yunnan \| GG \| \| SPM096 \| *C. sinensis* (L.) O. Kuntze var*. sinensis* \| Guizhou \| AG \| \| SPM097 \| *C. sinensis* var. *assamica* (Masters) Kitamura \| Guangdong \| AG \| \| SPM098 \| *C. sinensis* var. *assamica* (Masters) Kitamura \| Guangdong \| GG \| \| SPM099 \| *C. sinensis* var. *assamica* (Masters) Kitamura \| Guangdong \| GG \| \| SPM100 \| *C. sinensis* var. *assamica* (Masters) Kitamura \| Guangdong \| GG \| \| SPM101 \| *C. sinensis* var. *assamica* (Masters) Kitamura \| Yunnan \| GG \| \| SPM102 \| *C.* *sinensis* var. *pubilimba* Chang \| Guangdong \| GG \| \| SPM103 \| *C. sinensis* (L.) O. Kuntze var*. sinensis* \| Hubei \| GG \| \| SPM104 \| *C. sinensis* (L.) O. Kuntze var*. sinensis* \| Anhui \| AG \| \| SPM105 \| *C. sinensis* (L.) O. Kuntze var*. sinensis* \| Anhui \| GG \| \| SPM106 \| *C. sinensis* (L.) O. Kuntze var*. sinensis* \| Zhejiang \| GG \| \| SPM107 \| *C. sinensis* (L.) O. Kuntze var*. sinensis* \| Hunan \| GG \| \| SPM108 \| *C. sinensis* (L.) O. Kuntze var*. sinensis* \| Zhejiang \| AG \| \| SPM109 \| *C. sinensis* (L.) O. Kuntze var*. sinensis* \| Zhejiang \| GG \| \| SPM110 \| *C. sinensis* (L.) O. Kuntze var*. sinensis* \| Anhui \| AG \| \| SPM111 \| *C. sinensis* (L.) O. Kuntze var*. sinensis* \| Fujian \| GG \| \| SPM112 \| *C. sinensis* (L.) O. Kuntze var*. sinensis* \| Fujian \| GG \| \| SPM113 \| *C. sinensis* (L.) O. Kuntze var*. sinensis* \| Fujian \| AG \| \| SPM114 \| *C. sinensis* (L.) O. Kuntze var*. sinensis* \| Fujian \| GG \| \| SPM115 \| *C. sinensis* (L.) O. Kuntze var*. sinensis* \| Hunan \| GG \| \| SPM116 \| *C. sinensis* (L.) O. Kuntze var*. sinensis* \| Zhejiang \| GG \| \| SPM117 \| *C. sinensis* (L.) O. Kuntze var*. sinensis* \| Fujian \| GG \| \| SPM118 \| *C. sinensis* (L.) O. Kuntze var*. sinensis* \| Fujian \| GG \| \| SPM119 \| *C. sinensis* (L.) O. Kuntze var*. sinensis* \| Hunan \| AG \| \| SPM120 \| *C. sinensis* (L.) O. Kuntze var*. sinensis* \| Taiwan \| GG \| \| SPM121 \| *C. sinensis* (L.) O. Kuntze var*. sinensis* \| Fujian \| GG \| \| SPM122 \| *C. sinensis* (L.) O. Kuntze var*. sinensis* \| Fujian \| GG \| \| SPM123 \| *C. sinensis* (L.) O. Kuntze var*. sinensis* \| Zhejiang \| GG \| \| SPM124 \| *C. sinensis* (L.) O. Kuntze var*. sinensis* \| Guizhou \| AG \| \| SPM125 \| *C. sinensis* (L.) O. Kuntze var*. sinensis* \| Anhui \| GG \| \| SPM126 \| *C. sinensis* (L.) O. Kuntze var*. sinensis* \| Fujian \| GG \| \| SPM127 \| *C. sinensis* (L.) O. Kuntze var*. sinensis* \| Anhui \| GG \| \| SPM128 \| *C. sinensis* (L.) O. Kuntze var*. sinensis* \| Zhejiang \| GG \| \| SPM129 \| *C. sinensis* (L.) O. Kuntze var*. sinensis* \| Jiangsu \| GG \| \| SPM130 \| *C. sinensis* (L.) O. Kuntze var*. sinensis* \| Jiangsu \| GG \| \| SPM131 \| *C. sinensis* (L.) O. Kuntze var*. sinensis* \| Anhui \| GG \| \| SPM132 \| *C. sinensis* (L.) O. Kuntze var*. sinensis* \| Fujian \| GG \| \| SPM133 \| *C. sinensis* (L.) O. Kuntze var*. sinensis* \| Zhejiang \| AG \| \| SPM134 \| *C. sinensis* (L.) O. Kuntze var*. sinensis* \| Fujian \| GG \| \| SPM135 \| *C. sinensis* (L.) O. Kuntze var*. sinensis* \| Zhejiang \| GG \| \| SPM136 \| *C. sinensis* (L.) O. Kuntze var*. sinensis* \| Hunan \| GG \| \| SPM137 \| *C. sinensis* (L.) O. Kuntze var*. sinensis* \| Jiangxi \| AG \| \| SPM138 \| *C. sinensis* (L.) O. Kuntze var*. sinensis* \| Zhejiang \| GG \| \| SPM139 \| *C.* *sinensis* var. *pubilimba* Chang \| Guangxi \| GG \| \| SPM140 \| *C. sinensis* (L.) O. Kuntze var*. sinensis* \| Guangdong \| GG \| \| SPM141 \| *C. sinensis* (L.) O. Kuntze var*. sinensis* \| Chongqing \| GG \| \| SPM142 \| *C.* *sinensis* var. *pubilimba* Chang \| Guangxi \| AG \| \| SPM143 \| *C. sinensis* (L.) O. Kuntze var*. sinensis* \| Zhejiang \| AG \| \| SPM144 \| *C. sinensis* (L.) O. Kuntze var*. sinensis* \| Zhejiang \| AG \| \| SPM145 \| *C. sinensis* (L.) O. Kuntze var*. sinensis* \| Zhejiang \| AG \| \| SPM146 \| *C. sinensis* (L.) O. Kuntze var*. sinensis* \| Zhejiang \| AG \| \| SPM147 \| *C. sinensis* (L.) O. Kuntze var*. sinensis* \| Zhejiang \| AA \| \| SPM148 \| *C. sinensis* (L.) O. Kuntze var*. sinensis* \| Zhejiang \| AG \| \| SPM149 \| *C. sinensis* (L.) O. Kuntze var*. sinensis* \| Zhejiang \| AG \| \| SPM150 \| *C. sinensis* (L.) O. Kuntze var*. sinensis* \| Zhejiang \| AG \| \| SPM151 \| *C. sinensis* (L.) O. Kuntze var*. sinensis* \| Zhejiang \| GG \| \| SPM152 \| *C. sinensis* (L.) O. Kuntze var*. sinensis* \| Zhejiang \| AA \| \| SPM153 \| *C. sinensis* (L.) O. Kuntze var*. sinensis* \| Zhejiang \| AG \| \| SPM154 \| *C. sinensis* (L.) O. Kuntze var*. sinensis* \| Zhejiang \| AG \| \| SPM155 \| *C. sinensis* (L.) O. Kuntze var*. sinensis* \| Zhejiang \| AG \| \| SPM156 \| *C. sinensis* (L.) O. Kuntze var*. sinensis* \| Zhejiang \| AA \| \| SPM157 \| *C. sinensis* (L.) O. Kuntze var*. sinensis* \| Fujian \| GG \| \| SPM158 \| *C. sinensis* (L.) O. Kuntze var*. sinensis* \| Zhejiang \| AG \| \| SPM159 \| *C. sinensis* var. *assamica* (Masters) Kitamura \| Guangdong \| GG \| \| SPM160 \| *C. sinensis* (L.) O. Kuntze var*. sinensis* \| Zhejiang \| AG \| \| SPM161 \| *C. sinensis* (L.) O. Kuntze var*. sinensis* \| Fujian \| AG \| \| SPM162 \| *C. sinensis* (L.) O. Kuntze var*. sinensis* \| Fujian \| GG \| \| SPM163 \| *C. sinensis* (L.) O. Kuntze var*. sinensis* \| Fujian \| GG \| \| SPM164 \| *C. sinensis* (L.) O. Kuntze var*. sinensis* \| Zhejiang \| GG \| \| SPM165 \| *C. sinensis* (L.) O. Kuntze var*. sinensis* \| Zhejiang \| GG \| \| SPM166 \| *C. sinensis* (L.) O. Kuntze var*. sinensis* \| Taiwan \| GG \| \| SPM167 \| *C. sinensis* (L.) O. Kuntze var*. sinensis* \| Guizhou \| AG \| \| SPM168 \| *C. sinensis* (L.) O. Kuntze var*. sinensis* \| Anhui \| GG \| \| SPM169 \| *C. sinensis* (L.) O. Kuntze var*. sinensis* \| Anhui \| GG \| \| SPM170 \| *C. sinensis* (L.) O. Kuntze var*. sinensis* \| Zhejiang \| AG \| \| SPM171 \| *C. sinensis* (L.) O. Kuntze var*. sinensis* \| Zhejiang \| AG \| \| SPM172 \| *C. sinensis* (L.) O. Kuntze var*. sinensis* \| Zhejiang \| AG \| \| SPM173 \| *C. sinensis* (L.) O. Kuntze var*. sinensis* \| Zhejiang \| AG \| \| SPM174 \| *C. sinensis* (L.) O. Kuntze var*. sinensis* \| Zhejiang \| AG \| \| SPM175 \| *C. sinensis* (L.) O. Kuntze var*. sinensis* \| Zhejiang \| AG \| \| SPM176 \| *C. sinensis* (L.) O. Kuntze var*. sinensis* \| Zhejiang \| AA \|   Table S3 ANOVA between SNP556781 and TCC \| \| \| Genotype \| \| \| \| \| \| \| --- \| --- \| --- \| --- \| --- \| --- \| \|  \| Sum of Squares \| df \| Mean Square \| F \| Sig. \| \| Between Groups \| 44.944 \| 2 \| 22.472 \| 5.814 \| .004 \| \| Within Groups \| 668.710 \| 173 \| 3.865 \|  \|  \| \| Total \| 713.654 \| 175 \|  \|  \|  \|  \| TCC \| \| \| \| \| \| \| \| \| \| --- \| --- \| --- \| --- \| --- \| --- \| --- \| --- \| --- \| \| (I) Genotype \| \| (J)Genotype \| \| Mean Difference (I-J) \| Std. Error \| Sig. \| 95% Confidence Interval \| \| \| Lower Bound \| Upper Bound \| \|  \| AA \|  \| AG \| 1.43103 \| .62964 \| .024 \| .1883 \| 2.6738 \| \| GG \| 1.94583 \| .59718 \| .001 \| .7671 \| 3.1245 \| \| AG \|  \| AA \| -1.43103 \| .62964 \| .024 \| -2.6738 \| -.1883 \| \| GG \| .51481 \| .32992 \| .120 \| -.1364 \| 1.1660 \| \| GG \|  \| AA \| -1.94583 \| .59718 \| .001 \| -3.1245 \| -.7671 \| \| AG \| -.51481 \| .32992 \| .120 \| -1.1660 \| .1364 \| \| \|  \| |  |
| --- | --- | --- | --- | --- | --- | --- | --- | --- | --- | --- | --- | --- | --- | --- | --- | --- | --- | --- | --- | --- | --- | --- | --- | --- | --- | --- | --- | --- | --- | --- | --- | --- | --- | --- | --- | --- | --- | --- | --- | --- | --- | --- | --- | --- | --- | --- | --- | --- | --- | --- | --- | --- | --- | --- | --- | --- | --- | --- | --- | --- | --- | --- | --- | --- | --- | --- | --- | --- | --- | --- | --- | --- | --- | --- | --- | --- | --- | --- | --- | --- | --- | --- | --- | --- | --- | --- | --- | --- | --- | --- | --- | --- | --- | --- | --- | --- | --- | --- | --- | --- | --- | --- | --- | --- | --- | --- | --- | --- | --- | --- | --- | --- | --- | --- | --- | --- | --- | --- | --- | --- | --- | --- | --- | --- | --- | --- | --- | --- | --- | --- | --- | --- | --- | --- | --- | --- | --- | --- | --- | --- | --- | --- | --- | --- | --- | --- | --- | --- | --- | --- | --- | --- | --- | --- | --- | --- | --- | --- | --- | --- | --- | --- | --- | --- | --- | --- | --- | --- | --- | --- | --- | --- | --- | --- | --- | --- | --- | --- | --- | --- | --- | --- | --- | --- | --- | --- | --- | --- | --- | --- | --- | --- | --- | --- | --- | --- | --- | --- | --- | --- | --- | --- | --- | --- | --- | --- | --- | --- | --- | --- | --- | --- | --- | --- | --- | --- | --- | --- | --- | --- | --- | --- | --- | --- | --- | --- | --- | --- | --- | --- | --- | --- | --- | --- | --- | --- | --- | --- | --- | --- | --- | --- | --- | --- | --- | --- | --- | --- | --- | --- | --- | --- | --- | --- | --- | --- | --- | --- | --- | --- | --- | --- | --- | --- | --- | --- | --- | --- | --- | --- | --- | --- | --- | --- | --- | --- | --- | --- | --- | --- | --- | --- | --- | --- | --- | --- | --- | --- | --- | --- | --- | --- | --- | --- | --- | --- | --- | --- | --- | --- | --- | --- | --- | --- | --- | --- | --- | --- | --- | --- | --- | --- | --- | --- | --- | --- | --- | --- | --- | --- | --- | --- | --- | --- | --- | --- | --- | --- | --- | --- | --- | --- | --- | --- | --- | --- | --- | --- | --- | --- | --- | --- | --- | --- | --- | --- | --- | --- | --- | --- | --- | --- | --- | --- | --- | --- | --- | --- | --- | --- | --- | --- | --- | --- | --- | --- | --- | --- | --- | --- | --- | --- | --- | --- | --- | --- | --- | --- | --- | --- | --- | --- | --- | --- | --- | --- | --- | --- | --- | --- | --- | --- | --- | --- | --- | --- | --- | --- | --- | --- | --- | --- | --- | --- | --- | --- | --- | --- | --- | --- | --- | --- | --- | --- | --- | --- | --- | --- | --- | --- | --- | --- | --- | --- | --- | --- | --- | --- | --- | --- | --- | --- | --- | --- | --- | --- | --- | --- | --- | --- | --- | --- | --- | --- | --- | --- | --- | --- | --- | --- | --- | --- | --- | --- | --- | --- | --- | --- | --- | --- | --- | --- | --- | --- | --- | --- | --- | --- | --- | --- | --- | --- | --- | --- | --- | --- | --- | --- | --- | --- | --- | --- | --- | --- | --- | --- | --- | --- | --- | --- | --- | --- | --- | --- | --- | --- | --- | --- | --- | --- | --- | --- | --- | --- | --- | --- | --- | --- | --- | --- | --- | --- | --- | --- | --- | --- | --- | --- | --- | --- | --- | --- | --- | --- | --- | --- | --- | --- | --- | --- | --- | --- | --- | --- | --- | --- | --- | --- | --- | --- | --- | --- | --- | --- | --- | --- | --- | --- | --- | --- | --- | --- | --- | --- | --- | --- | --- | --- | --- | --- | --- | --- | --- | --- | --- | --- | --- | --- | --- | --- | --- | --- | --- | --- | --- | --- | --- | --- | --- | --- | --- | --- | --- | --- | --- | --- | --- | --- | --- | --- | --- | --- | --- | --- | --- | --- | --- | --- | --- | --- | --- | --- | --- | --- | --- | --- | --- | --- | --- | --- | --- | --- | --- | --- | --- | --- | --- | --- | --- | --- | --- | --- | --- | --- | --- | --- | --- | --- | --- | --- | --- | --- | --- | --- | --- | --- | --- | --- | --- | --- | --- | --- | --- | --- | --- | --- | --- | --- | --- | --- | --- | --- | --- | --- | --- | --- | --- | --- | --- | --- | --- | --- | --- | --- | --- | --- | --- | --- | --- | --- | --- | --- | --- | --- | --- | --- | --- | --- | --- | --- | --- | --- | --- | --- | --- | --- | --- | --- | --- | --- | --- | --- | --- | --- | --- | --- | --- | --- | --- | --- | --- | --- | --- | --- | --- | --- | --- | --- | --- | --- | --- | --- | --- | --- | --- | --- | --- | --- | --- | --- | --- | --- | --- | --- | --- | --- | --- | --- | --- | --- | --- | --- | --- | --- | --- | --- | --- | --- | --- | --- | --- | --- | --- | --- | --- | --- | --- | --- | --- | --- | --- | --- | --- | --- | --- | --- | --- | --- | --- | --- | --- | --- | --- | --- | --- | --- | --- | --- | --- | --- | --- | --- | --- | --- | --- | --- | --- | --- | --- | --- | --- | --- | --- | --- | --- | --- | --- | --- | --- | --- | --- | --- | --- | --- | --- | --- | --- | --- | --- | --- | --- | --- | --- | --- | --- | --- | --- | --- | --- | --- | --- | --- | --- | --- | --- | --- | --- | --- | --- | --- | --- | --- | --- | --- | --- | --- | --- | --- | --- | --- | --- | --- | --- | --- | --- | --- | --- | --- | --- | --- | --- | --- | --- | --- | --- | --- | --- | --- | --- | --- | --- | --- | --- | --- | --- | --- | --- | --- | --- | --- | --- | --- | --- | --- | --- | --- | --- | --- | --- | --- | --- | --- | --- | --- | --- | --- | --- | --- | --- | --- | --- | --- | --- | --- | --- | --- | --- | --- | --- | --- | --- | --- | --- | --- | --- | --- | --- | --- | --- | --- | --- | --- | --- | --- | --- | --- | --- | --- | --- | --- | --- | --- | --- | --- | --- | --- | --- | --- | --- | --- | --- | --- | --- | --- | --- | --- | --- | --- | --- | --- | --- | --- | --- | --- | --- | --- | --- | --- | --- | --- | --- | --- | --- | --- | --- | --- | --- | --- | --- | --- | --- | --- | --- | --- | --- | --- | --- | --- | --- | --- | --- | --- | --- | --- | --- | --- | --- | --- | --- | --- | --- | --- | --- | --- | --- | --- | --- | --- | --- | --- | --- | --- | --- | --- | --- | --- | --- | --- | --- | --- | --- | --- | --- | --- | --- | --- | --- | --- | --- | --- | --- | --- | --- | --- | --- | --- | --- | --- | --- |
| Table S4 The genotype (SNP556781) and catechin of six tea samples |  |
| \| Sample \| Genotype \|  \| EGCG(%) \| EGC(%) \| ECG(%) \| EC(%) \| TCC(%) \|  \| \| --- \| --- \| --- \| --- \| --- \| --- \| --- \| --- \| --- \| \| YS \| AG \|  \| 10.83 \| 1.09 \| 2.09 \| 0.54 \| 14.55 \|  \| \| BD \| AG \|  \| 10.35 \| 1.76 \| 4.20 \| 1.57 \| 17.89 \|  \| \| F1-1 \| AG \|  \| 14.77 \| 2.10 \| 2.33 \| 0.66 \| 19.86 \|  \| \| F1-2 \| AG \|  \| 8.88 \| 1.67 \| 2.89 \| 0.90 \| 14.34 \|  \| \| F1-3 \| AG \|  \| 11.22 \| 1.76 \| 2.00 \| 0.66 \| 15.65 \|  \| \| F1-4 \| AG \|  \| 9.67 \| 2.31 \| 3.16 \| 0.96 \| 16.10 \|  \| |  |

Table S5 Catechin of 176 tea accessions

| Number | EGCG(%) | EGC(%) | ECG(%) | EC(%) | TCC(%) |
| --- | --- | --- | --- | --- | --- |
| SPM001 | 8.64 | 1.09 | 2.46 | 0.55 | 12.74 |
| SPM002 | 8.20 | 0.75 | 2.01 | 0.41 | 11.37 |
| SPM003 | 11.28 | 2.10 | 2.60 | 0.85 | 16.83 |
| SPM004 | 11.24 | 2.26 | 2.12 | 0.78 | 16.40 |
| SPM005 | 9.43 | 1.51 | 2.95 | 0.81 | 14.70 |
| SPM006 | 10.25 | 1.93 | 3.66 | 0.88 | 16.72 |
| SPM007 | 8.94 | 2.00 | 4.39 | 0.99 | 16.32 |
| SPM008 | 10.59 | 2.05 | 2.16 | 0.68 | 15.48 |
| SPM009 | 9.02 | 1.81 | 3.45 | 0.85 | 15.13 |
| SPM010 | 9.10 | 1.42 | 2.33 | 0.60 | 13.45 |
| SPM011 | 10.46 | 2.17 | 2.35 | 0.73 | 15.71 |
| SPM012 | 10.90 | 2.05 | 2.77 | 0.98 | 16.70 |
| SPM013 | 10.70 | 1.01 | 3.62 | 0.70 | 16.03 |
| SPM014 | 10.32 | 1.43 | 2.85 | 0.56 | 15.16 |
| SPM015 | 8.42 | 1.81 | 3.78 | 1.02 | 15.03 |
| SPM016 | 10.19 | 1.96 | 2.40 | 0.67 | 15.22 |
| SPM017 | 9.18 | 0.95 | 2.63 | 0.35 | 13.11 |
| SPM018 | 7.89 | 1.26 | 2.45 | 0.75 | 12.35 |
| SPM019 | 8.46 | 1.40 | 2.84 | 0.83 | 13.53 |
| SPM020 | 10.26 | 1.53 | 2.06 | 0.64 | 14.49 |
| SPM021 | 9.63 | 2.00 | 2.59 | 0.73 | 14.95 |
| SPM022 | 7.56 | 1.73 | 2.67 | 0.62 | 12.58 |
| SPM023 | 8.72 | 1.85 | 2.03 | 0.61 | 13.21 |
| SPM024 | 8.13 | 1.71 | 2.57 | 0.81 | 13.22 |
| SPM025 | 8.56 | 1.97 | 3.10 | 1.02 | 14.65 |
| SPM026 | 8.33 | 1.59 | 3.11 | 0.79 | 13.82 |
| SPM027 | 10.23 | 2.16 | 2.35 | 0.77 | 15.51 |
| SPM028 | 9.41 | 2.06 | 2.26 | 0.87 | 14.60 |
| SPM029 | 8.71 | 0.88 | 2.19 | 0.48 | 12.26 |
| SPM030 | 11.96 | 1.32 | 3.98 | 0.65 | 17.91 |
| SPM031 | 11.32 | 1.13 | 3.17 | 0.59 | 16.21 |
| SPM032 | 10.12 | 1.02 | 2.71 | 0.36 | 14.21 |
| SPM033 | 8.43 | 3.33 | 3.87 | 1.73 | 17.36 |
| SPM034 | 9.09 | 2.33 | 2.91 | 1.13 | 15.46 |
| SPM035 | 9.19 | 1.86 | 2.78 | 0.80 | 14.63 |
| SPM036 | 8.58 | 1.31 | 3.87 | 0.74 | 14.50 |
| SPM037 | 10.23 | 1.33 | 2.43 | 0.53 | 14.52 |
| SPM038 | 8.30 | 1.41 | 3.08 | 0.74 | 13.53 |
| SPM039 | 9.51 | 1.06 | 3.16 | 0.50 | 14.23 |
| SPM040 | 8.40 | 2.16 | 2.46 | 1.01 | 14.03 |
| SPM041 | 8.66 | 1.29 | 2.18 | 0.67 | 12.80 |
| SPM042 | 8.59 | 1.59 | 3.39 | 1.12 | 14.69 |
| SPM043 | 7.71 | 1.80 | 2.34 | 0.58 | 12.43 |
| SPM044 | 9.33 | 1.54 | 2.19 | 0.68 | 13.74 |
| SPM045 | 9.01 | 1.57 | 2.44 | 0.70 | 13.72 |
| SPM046 | 8.79 | 1.53 | 2.86 | 0.73 | 13.91 |
| SPM047 | 8.64 | 1.13 | 3.51 | 0.57 | 13.85 |
| SPM048 | 8.04 | 1.18 | 3.53 | 0.88 | 13.63 |
| SPM049 | 9.93 | 1.55 | 3.88 | 0.91 | 16.27 |
| SPM050 | 7.53 | 1.27 | 2.44 | 0.69 | 11.93 |
| SPM051 | 10.55 | 1.42 | 1.99 | 0.53 | 14.49 |
| SPM052 | 9.45 | 1.25 | 2.98 | 0.74 | 14.42 |
| SPM053 | 9.89 | 1.71 | 3.25 | 0.91 | 15.76 |
| SPM054 | 10.83 | 1.09 | 2.09 | 0.54 | 14.55 |
| SPM055 | 8.34 | 1.13 | 3.28 | 0.65 | 13.40 |
| SPM056 | 9.50 | 1.80 | 2.80 | 0.78 | 14.88 |
| SPM057 | 6.61 | 1.09 | 4.00 | 0.85 | 12.55 |
| SPM058 | 7.90 | 1.77 | 2.95 | 1.16 | 13.78 |
| SPM059 | 9.54 | 1.58 | 2.32 | 0.72 | 14.16 |
| SPM060 | 9.06 | 1.59 | 3.84 | 0.92 | 15.41 |
| SPM061 | 8.09 | 1.62 | 2.71 | 0.71 | 13.13 |
| SPM062 | 8.74 | 1.77 | 3.43 | 1.03 | 14.97 |
| SPM063 | 8.91 | 1.38 | 2.29 | 0.70 | 13.28 |
| SPM064 | 7.36 | 1.10 | 2.46 | 0.67 | 11.59 |
| SPM065 | 8.34 | 1.36 | 2.60 | 0.78 | 13.08 |
| SPM066 | 7.07 | 1.37 | 3.33 | 0.91 | 12.68 |
| SPM067 | 10.86 | 1.39 | 3.14 | 0.62 | 16.01 |
| SPM068 | 10.61 | 1.15 | 3.51 | 0.62 | 15.89 |
| SPM069 | 13.75 | 2.54 | 4.74 | 1.38 | 22.41 |
| SPM070 | 12.80 | 1.46 | 3.65 | 0.68 | 18.59 |
| SPM071 | 11.18 | 1.70 | 3.80 | 0.78 | 17.46 |
| SPM072 | 8.52 | 2.22 | 3.96 | 1.24 | 15.94 |
| SPM073 | 8.76 | 1.14 | 3.79 | 1.26 | 14.95 |
| SPM074 | 10.39 | 2.74 | 1.64 | 0.70 | 15.47 |
| SPM075 | 8.78 | 1.43 | 5.26 | 1.30 | 16.77 |
| SPM076 | 8.02 | 1.94 | 3.05 | 1.34 | 14.35 |
| SPM077 | 8.05 | 1.33 | 5.60 | 1.21 | 16.19 |
| SPM078 | 9.31 | 2.02 | 2.72 | 0.91 | 14.96 |
| SPM079 | 8.36 | 2.25 | 3.53 | 1.22 | 15.36 |
| SPM080 | 9.90 | 1.03 | 3.66 | 0.54 | 15.13 |
| SPM081 | 6.10 | 1.26 | 3.39 | 1.43 | 12.18 |
| SPM082 | 7.04 | 1.21 | 5.06 | 1.39 | 14.70 |
| SPM083 | 8.43 | 1.81 | 2.50 | 1.04 | 13.78 |
| SPM084 | 7.10 | 1.64 | 5.31 | 1.36 | 15.41 |
| SPM085 | 10.14 | 2.32 | 4.20 | 1.18 | 17.84 |
| SPM086 | 9.34 | 1.55 | 5.04 | 1.02 | 16.95 |
| SPM087 | 8.96 | 2.11 | 2.52 | 1.10 | 14.69 |
| SPM088 | 11.33 | 1.65 | 3.42 | 0.94 | 17.34 |
| SPM089 | 8.37 | 1.34 | 7.28 | 1.53 | 18.52 |
| SPM090 | 9.59 | 1.45 | 1.74 | 0.42 | 13.20 |
| SPM091 | 10.81 | 1.35 | 2.53 | 0.71 | 15.40 |
| SPM092 | 6.55 | 1.21 | 4.33 | 1.08 | 13.17 |
| SPM093 | 9.28 | 1.10 | 3.22 | 0.79 | 14.39 |
| SPM094 | 7.20 | 2.22 | 3.84 | 2.51 | 15.77 |
| SPM095 | 10.57 | 2.17 | 3.01 | 1.16 | 16.91 |
| SPM096 | 8.38 | 2.63 | 4.90 | 1.59 | 17.50 |
| SPM097 | 8.96 | 1.76 | 4.10 | 1.26 | 16.08 |
| SPM098 | 11.32 | 1.17 | 4.67 | 0.62 | 17.78 |
| SPM099 | 9.87 | 0.72 | 3.22 | 0.33 | 14.14 |
| SPM100 | 8.17 | 1.72 | 4.56 | 0.92 | 15.37 |
| SPM101 | 13.57 | 2.24 | 3.01 | 0.86 | 19.68 |
| SPM102 | 7.72 | 0.53 | 2.53 | 0.47 | 11.25 |
| SPM103 | 8.32 | 2.05 | 2.82 | 1.10 | 14.29 |
| SPM104 | 8.17 | 1.00 | 2.25 | 0.55 | 11.97 |
| SPM105 | 9.73 | 1.44 | 3.11 | 0.76 | 15.04 |
| SPM106 | 7.99 | 1.77 | 1.38 | 0.63 | 11.77 |
| SPM107 | 9.44 | 1.58 | 3.69 | 1.03 | 15.74 |
| SPM108 | 7.41 | 1.99 | 2.22 | 0.88 | 12.50 |
| SPM109 | 9.88 | 1.02 | 2.66 | 0.46 | 14.02 |
| SPM110 | 9.42 | 2.05 | 2.86 | 0.97 | 15.30 |
| SPM111 | 9.28 | 1.36 | 2.98 | 0.73 | 14.35 |
| SPM112 | 7.92 | 1.33 | 2.57 | 0.86 | 12.68 |
| SPM113 | 8.86 | 1.69 | 2.95 | 0.84 | 14.34 |
| SPM114 | 6.63 | 1.86 | 2.75 | 1.17 | 12.41 |
| SPM115 | 10.32 | 1.91 | 2.53 | 0.89 | 15.65 |
| SPM116 | 10.12 | 2.30 | 3.64 | 1.18 | 17.24 |
| SPM117 | 7.61 | 1.07 | 2.81 | 0.72 | 12.21 |
| SPM118 | 7.62 | 1.45 | 2.53 | 0.91 | 12.51 |
| SPM119 | 8.18 | 2.02 | 2.87 | 0.88 | 13.95 |
| SPM120 | 8.33 | 1.60 | 1.69 | 0.52 | 12.14 |
| SPM121 | 9.90 | 2.11 | 2.70 | 0.70 | 15.41 |
| SPM122 | 8.84 | 1.56 | 3.34 | 0.85 | 14.59 |
| SPM123 | 8.01 | 1.40 | 3.33 | 0.78 | 13.52 |
| SPM124 | 7.58 | 1.27 | 3.58 | 0.84 | 13.27 |
| SPM125 | 6.87 | 1.17 | 3.04 | 0.67 | 11.75 |
| SPM126 | 8.81 | 1.46 | 3.06 | 0.70 | 14.03 |
| SPM127 | 9.55 | 2.29 | 2.01 | 0.65 | 14.50 |
| SPM128 | 7.10 | 1.73 | 3.02 | 1.14 | 12.99 |
| SPM129 | 8.17 | 2.08 | 3.45 | 1.13 | 14.83 |
| SPM130 | 8.63 | 1.36 | 2.60 | 0.63 | 13.22 |
| SPM131 | 9.63 | 0.99 | 2.55 | 0.37 | 13.54 |
| SPM132 | 10.97 | 1.88 | 2.09 | 0.57 | 15.51 |
| SPM133 | 8.24 | 1.16 | 2.87 | 0.70 | 12.97 |
| SPM134 | 8.94 | 1.49 | 3.02 | 0.67 | 14.12 |
| SPM135 | 8.41 | 1.74 | 2.21 | 0.65 | 13.01 |
| SPM136 | 9.65 | 2.33 | 2.27 | 1.23 | 15.48 |
| SPM137 | 9.68 | 0.84 | 2.69 | 0.42 | 13.63 |
| SPM138 | 9.23 | 1.60 | 2.59 | 0.83 | 14.25 |
| SPM139 | 10.65 | 0.96 | 2.99 | 0.38 | 14.98 |
| SPM140 | 9.79 | 1.64 | 2.01 | 0.86 | 14.30 |
| SPM141 | 9.02 | 0.97 | 3.63 | 0.57 | 14.19 |
| SPM142 | 10.35 | 1.76 | 4.20 | 1.57 | 17.89 |
| SPM143 | 14.77 | 2.10 | 2.33 | 0.66 | 19.86 |
| SPM144 | 8.88 | 1.67 | 2.89 | 0.90 | 14.34 |
| SPM145 | 11.22 | 1.76 | 2.00 | 0.66 | 15.65 |
| SPM146 | 9.67 | 2.31 | 3.16 | 0.96 | 16.10 |
| SPM147 | 13.53 | 1.70 | 1.62 | 0.43 | 17.28 |
| SPM148 | 8.54 | 1.26 | 4.02 | 0.88 | 14.70 |
| SPM149 | 14.82 | 3.40 | 5.99 | 1.87 | 26.08 |
| SPM150 | 12.68 | 1.11 | 1.65 | 0.35 | 15.80 |
| SPM151 | 8.25 | 1.82 | 3.33 | 1.33 | 14.73 |
| SPM152 | 13.35 | 2.34 | 1.66 | 0.64 | 17.99 |
| SPM153 | 10.83 | 1.77 | 1.39 | 0.44 | 14.43 |
| SPM154 | 12.72 | 1.86 | 1.58 | 0.50 | 16.66 |
| SPM155 | 9.55 | 1.00 | 3.59 | 1.34 | 15.48 |
| SPM156 | 9.98 | 1.66 | 4.64 | 0.94 | 17.23 |
| SPM157 | 8.46 | 1.40 | 2.84 | 0.83 | 13.53 |
| SPM158 | 10.83 | 1.09 | 2.09 | 0.54 | 14.55 |
| SPM159 | 8.17 | 1.72 | 4.56 | 0.92 | 15.37 |
| SPM160 | 7.41 | 1.99 | 2.22 | 0.88 | 12.50 |
| SPM161 | 7.92 | 1.33 | 2.57 | 0.86 | 12.68 |
| SPM162 | 6.63 | 1.86 | 2.75 | 1.17 | 12.41 |
| SPM163 | 8.81 | 1.46 | 3.06 | 0.70 | 14.03 |
| SPM164 | 7.10 | 1.73 | 3.02 | 1.14 | 12.99 |
| SPM165 | 10.12 | 2.30 | 3.64 | 1.18 | 17.24 |
| SPM166 | 8.33 | 1.60 | 1.69 | 0.52 | 12.14 |
| SPM167 | 7.58 | 1.27 | 3.58 | 0.84 | 13.27 |
| SPM168 | 6.87 | 1.17 | 3.04 | 0.67 | 11.75 |
| SPM169 | 9.63 | 0.99 | 2.55 | 0.37 | 13.54 |
| SPM170 | 14.77 | 2.10 | 2.33 | 0.66 | 19.86 |
| SPM171 | 8.88 | 1.67 | 2.89 | 0.90 | 14.34 |
| SPM172 | 11.22 | 1.76 | 2.00 | 0.66 | 15.65 |
| SPM173 | 9.67 | 2.31 | 3.16 | 0.96 | 16.10 |
| SPM174 | 12.68 | 1.11 | 1.65 | 0.35 | 15.80 |
| SPM175 | 10.83 | 1.77 | 1.39 | 0.44 | 14.43 |
| SPM176 | 9.98 | 1.66 | 4.64 | 0.94 | 17.23 |


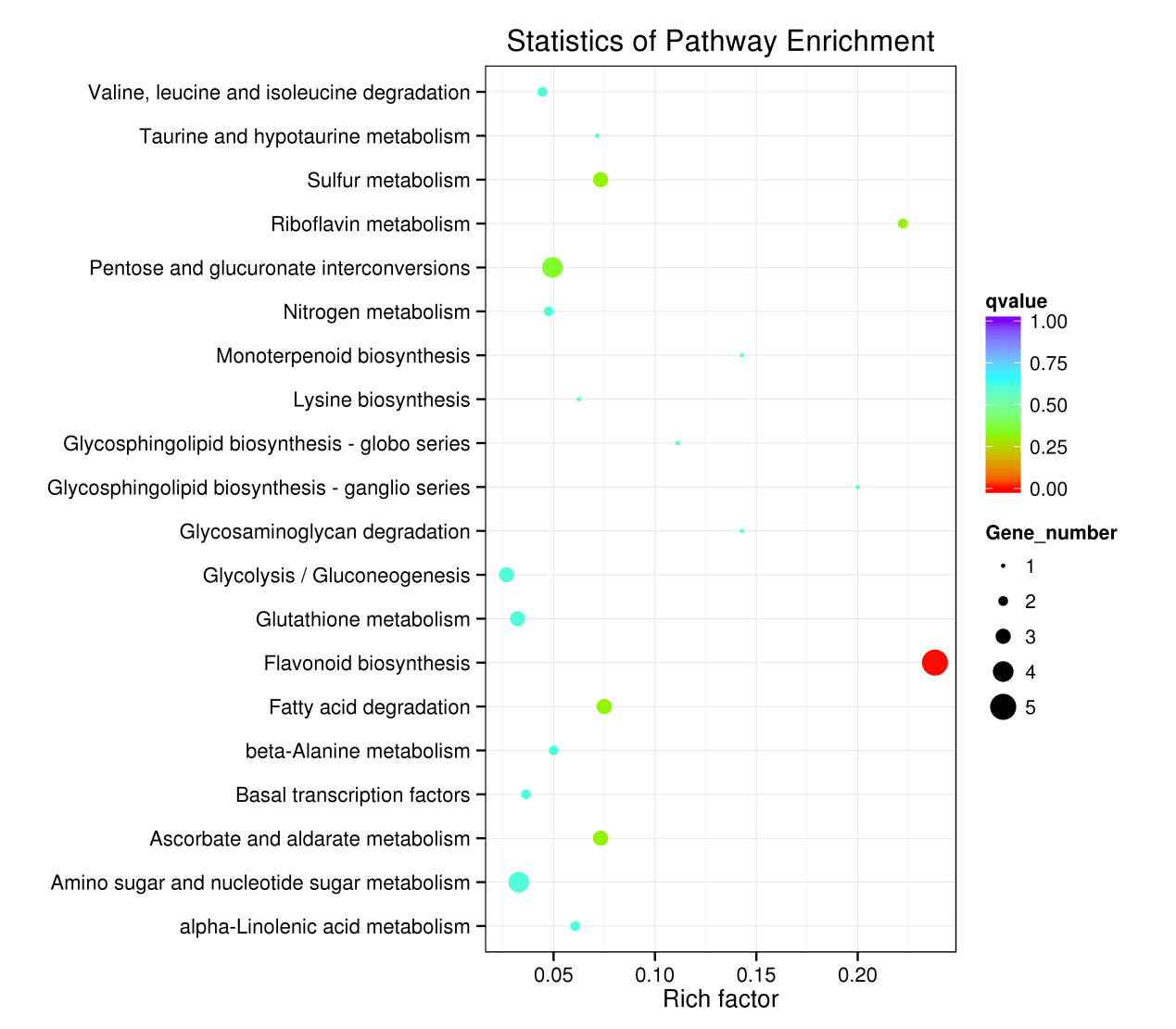


Figure S1 F1-2 vs BD up DEG enriched in KEGG pathway


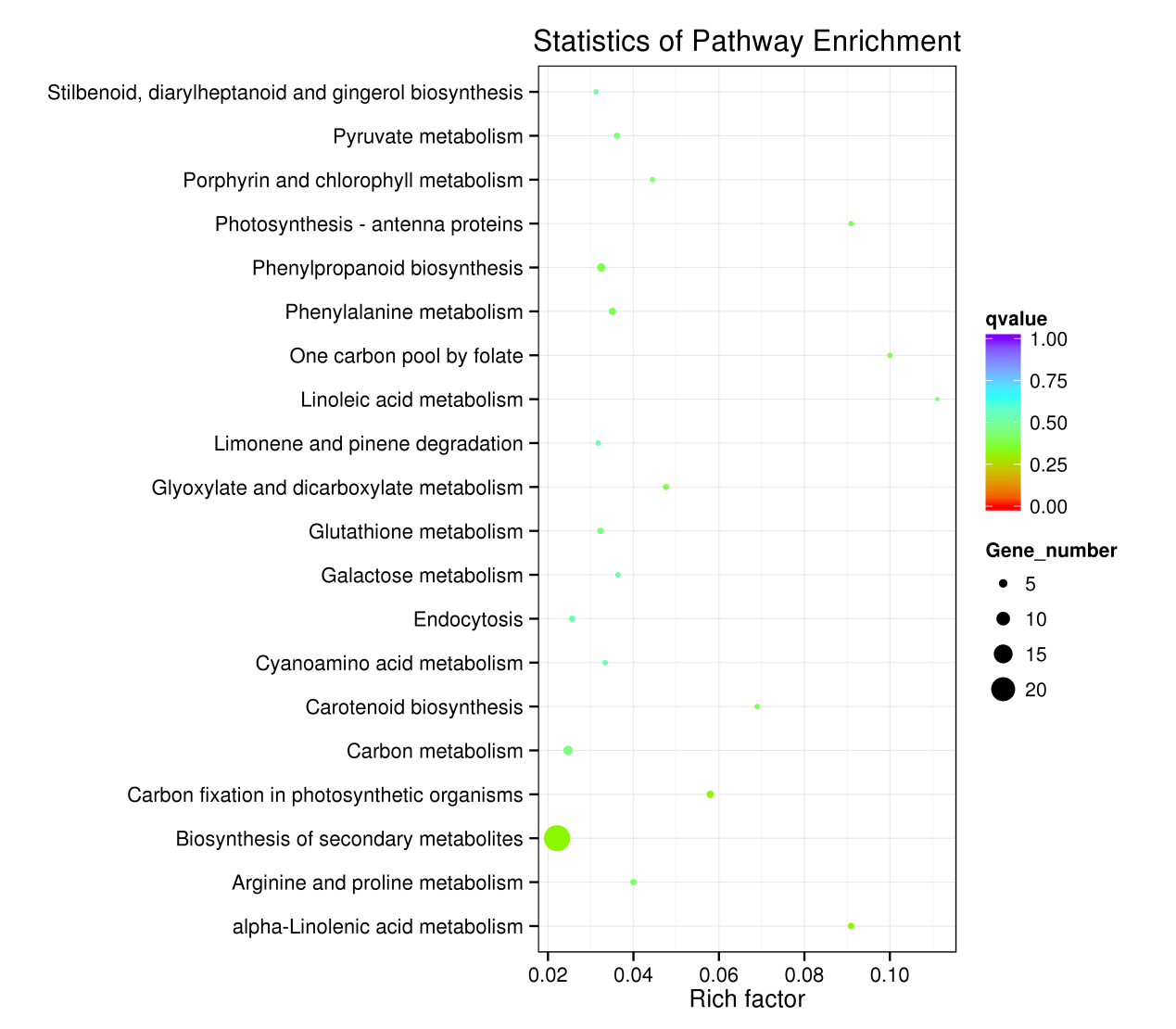


Figure S2 BD vsYS_up.DEG enriched in KEGG pathway


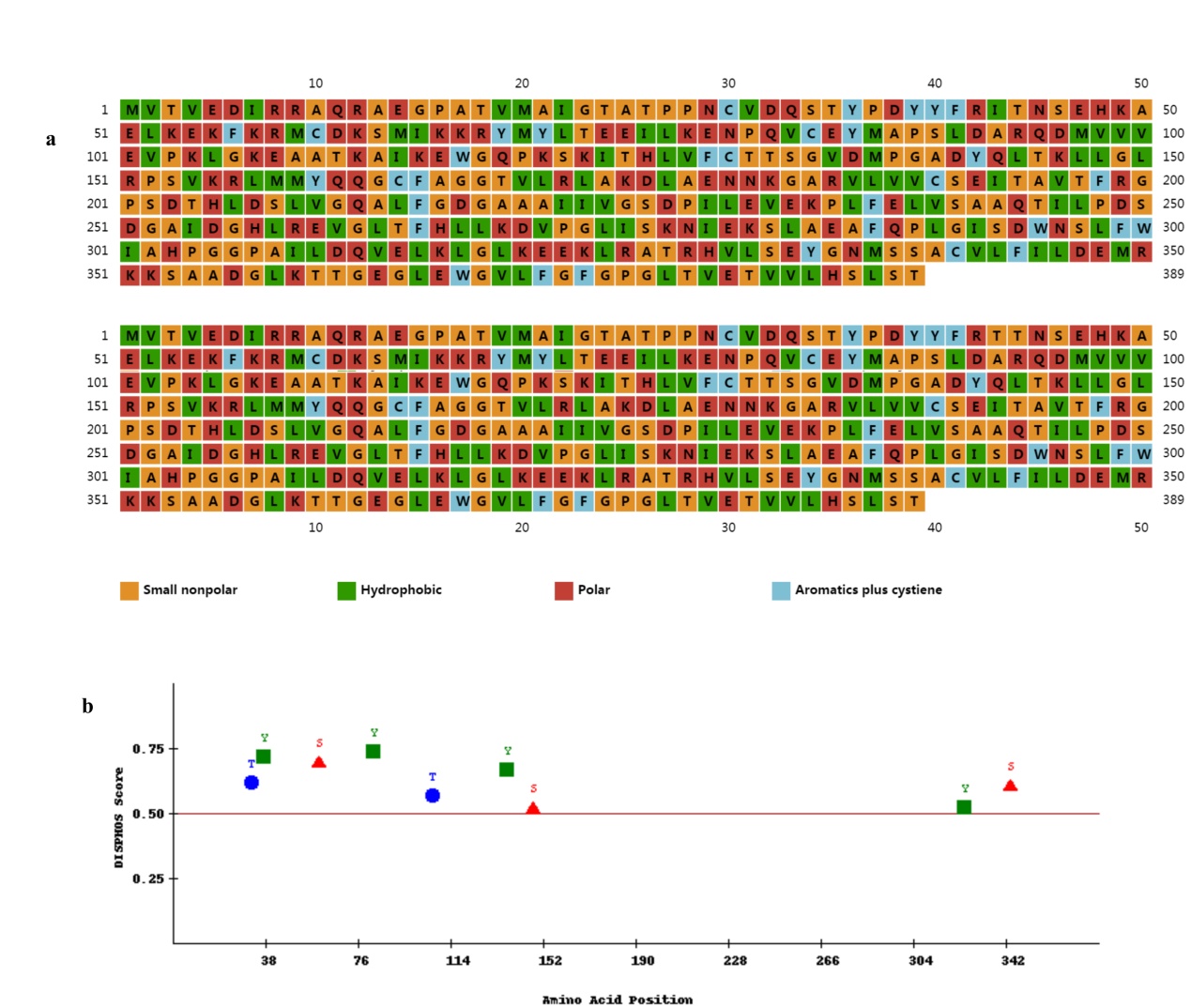


Figure S3 Amino acids coded by TEA023340.1
